# Rescue of neonatal cardiac dysfunction in mice by administration of cardiac progenitor cells *in utero*

**DOI:** 10.1038/ncomms9825

**Published:** 2015-11-23

**Authors:** Xiaoli Liu, Sean R. R. Hall, Zhihong Wang, He Huang, Sailaja Ghanta, Moises Di Sante, Annarosa Leri, Piero Anversa, Mark A. Perrella

**Affiliations:** 1 Division of Pulmonary and Critical Care Medicine, Department of Medicine, Brigham and Women's Hospital and Harvard Medical School, 75 Francis Street, Boston, Massachusetts 02115, USA; 2 Department of Pediatric Newborn Medicine, Brigham and Women's Hospital and Harvard Medical School, 75 Francis Street, Boston, Massachusetts 02115, USA; 3 Cardiovascular Medicine, Department of Medicine, Brigham and Women's Hospital and Harvard Medical School, 75 Francis Street, Boston, Massachusetts 02115, USA; 4 Department of Anesthesia, Brigham and Women's Hospital and Harvard Medical School, 75 Francis Street, Boston, Massachusetts 02115, USA

## Abstract

Striated preferentially expressed gene (Speg) is a member of the myosin light chain kinase family. We previously showed that disruption of the *Speg* gene locus in mice leads to a dilated cardiomyopathy with immature-appearing cardiomyocytes. Here we show that cardiomyopathy of *Speg*^*−/−*^ mice arises as a consequence of defects in cardiac progenitor cell (CPC) function, and that neonatal cardiac dysfunction can be rescued by *in utero* injections of wild-type CPCs into *Speg*^*−/−*^ foetal hearts. CPCs harvested from *Speg*^*−/−*^ mice display defects in clone formation, growth and differentiation into cardiomyocytes *in vitro*, which are associated with cardiac dysfunction *in vivo*. *In utero* administration of wild-type CPCs into the hearts of *Speg*^*−/−*^ mice results in CPC engraftment, differentiation and myocardial maturation, which rescues *Speg*^*−/−*^ mice from neonatal heart failure and increases the number of live births by fivefold. We propose that *in utero* administration of CPCs may have future implications for treatment of neonatal heart diseases.

## Abstract (web summary)

The protein Speg is expressed in the developing mouse heart, where its absence leads to neonatal cardiac disease. Here the authors trace the cardiomyopathy of Speg KO mice back to defects in cardiac progenitor cells (CPCs) and rescue it with injections of wild type CPCs into the foetal heart.

## Graphical abstract

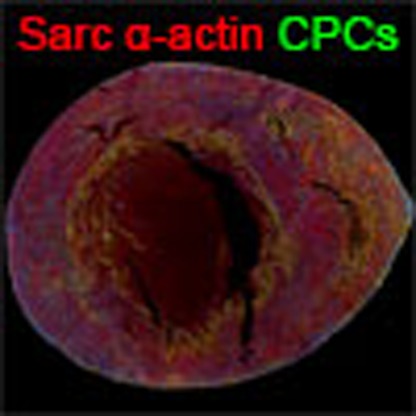

Congenital heart disease is the most common congenital disorder in newborns, and the most frequent cause of infant death from birth defects^1^. In addition to these structural defects of the heart, cardiomyopathies are also a significant cause of heart failure in children^2^. The sarcomere is the functional unit of striated muscle that generates contraction, and mutations in sarcomeric proteins may lead to either dilated or hypertrophic cardiomyopathies^3^,^4^,^5^,^6^,^7^. More specifically, mutations of proteins localized to the Z-disc, which defines the lateral border of the sarcomere, are involved in a variety of human cardiomyopathies^5^. We have previously shown that mutation of the striated preferentially expressed gene (*Speg*) in mice, which co-localizes with desmin at the Z-disc and is part of a muscle-specific gene locus^8^,^9^, leads to a dilated cardiomyopathy in mice^10^. Interestingly, mutations in the gene encoding *Speg* have recently been found in patients with centronucelar myopathy, a congenital skeletal muscle condition, and these patients additionally present with a dilated cardiomyopathy^11^.

The *Speg* gene locus, through alternative promoter use and splicing in a tissue-specific manner, generates four different isoforms^8^. Spegα and Spegβ are expressed specifically in striated muscle, and these two isoforms along with obscurin are unique members of the myosin light chain kinase (MLCK) family, containing two tandemly arranged serine/threonine kinase (MLCK) domains^12^. We have previously shown that during development, Speg isoforms are expressed predominantly in the heart throughout the first 18.5 days-post coitum (dpc)^10^. At that point in time, *Speg* mutant hearts have already begun to enlarge and exhibited marked cardiac dysfunction. Furthermore, we demonstrated that in *Speg* mutant hearts, there was evidence for decreased phosphorylation of α-tropomyosin^10^, a protein that resides in the thin filament of the sarcomere, and anchors at the Z-disc. Mutations in α-tropomyosin have also been found in patients with familial dilated cardiomyopathy^13^,^14^,^15^. It is known that the Z-disc is functionally important for more than just structural stability and force transmission, but also for cell signalling^5^. Our data suggest that like other sarcomeric Z-disc proteins, mutation of *Speg* may lead to more than just a structural abnormality of the sarcomere, and potentially has additional consequences leading to abnormal function of cardiomyocytes.

When assessing the structural organization of the *Speg* mutant (*Speg*^*−/−*^) hearts at 18.5 dpc, we found a reduced density of myocyte nuclei per unit area of tissue, in the absence of increased myocyte death^10^. Also, *Speg*^*−/−*^ hearts were composed of myocytes that were 20% larger than wild-type (*Speg*^*+/+*^) myocytes. These data suggested that mutation of the *Speg* gene locus altered the generation of cardiac parenchymal cells, particularly cardiomyocytes, during development. Electron microscopy of *Speg* mutant hearts at 18.5 dpc revealed evidence of myofibril disarray^10^. While this may be seen in failing hearts^16^, the thin, loosely arranged and less organized myofibrils of the *Speg* mutant hearts also depict a less mature myocyte^17^,^18^,^19^ compared with wild-type hearts. Of note, we previously demonstrated an increased expression of Spegα during differentiation of C2C12 myoblasts into myotubes^8^. While we proposed at the time that Spegα might serve as a sensitive marker of striated muscle differentiation, it is also feasible that expression of Speg isoforms may be important for cellular commitment and maturation.

Since the *Speg* mutant hearts have an abnormality in the generation of cardiomyocytes^10^, and expression of Speg isoforms have been associated with differentiation of striated muscle cells^8^, we speculated that Speg may be important for cardiac progenitor cell (CPC) function. It has been suggested previously that the formation of cardiomyocytes from the differentiation of CPCs is critical for cardiac growth during development^20^. While a recent mouse lineage tracing study challenged the importance of c-kit-positive CPCs in the regulation of cardiomyocyte renewal^21^, further investigations need to be performed^22^. Nevertheless, the multipotency and regenerative capabilities of harvested c-kit-positive CPCs have been extensively studied and confirmed by numerous laboratories^23^. In fact, the lineage tracing mice confirmed the presence of endogenous c-kit-positive cells that produce cardiomyocytes in the mouse heart^21^. A predominant fraction of CPCs in embryonic, foetal and neonatal hearts are positive for c-kit, and have the properties of clonogenicity, self-renewal and multipotency^20^,^24^,^25^,^26^. Thus, in the present study we wanted to determine whether c-kit-positive CPCs harvested from *Speg* mutant mice have functional abnormalities, and to determine whether administration of wild-type CPCs *in utero* could rescue the cardiac phenotype of *Speg* mutant mice. *Speg*^*−/−*^ CPCs revealed defects in clone formation, growth and differentiation into cardiomyocytes *in vitro*, and were associated with cardiac dysfunction *in vivo*. Administration of wild-type CPCs into the hearts of *Speg*^−/−^ foetuses resulted in CPC engraftment, differentiation and myocardial maturation, which rescued *Speg*^*−/−*^ mice from neonatal heart failure. These data suggest that *in utero* administration of CPCs may have future implications for treatment of neonatal heart diseases.

## Results

### Immature myocytes in the hearts of *Speg* mutant mice

**Figure 1:**
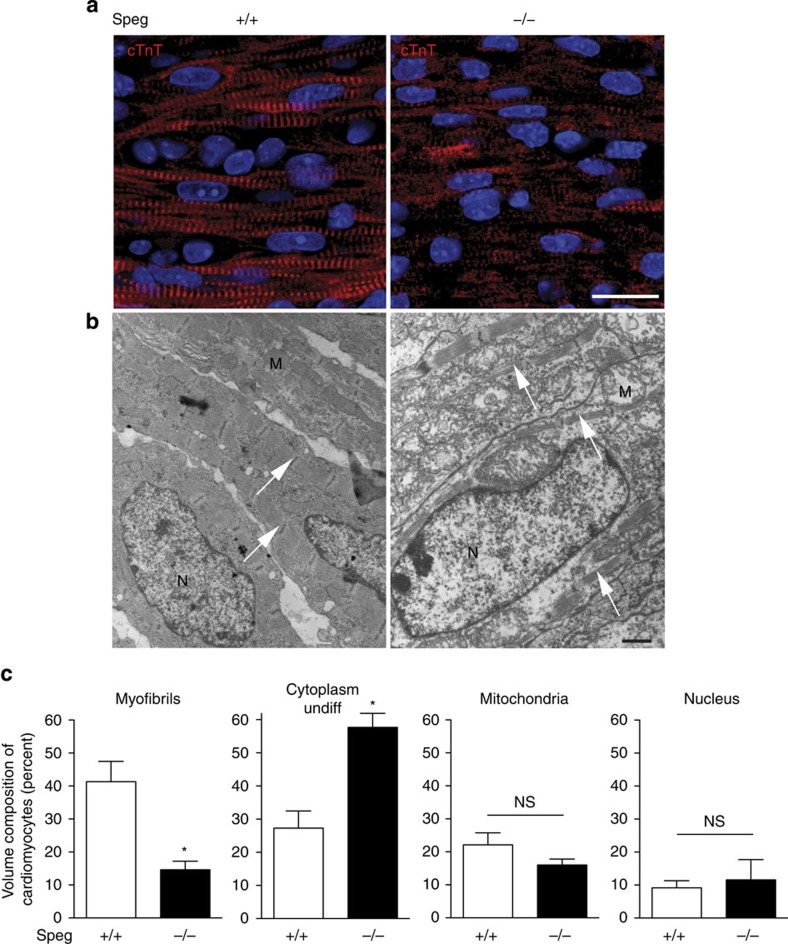
Structure of *Speg*^*−/−*^ hearts. (**a**) Confocal imaging of cardiac troponin T (cTnT) staining (red) of myocytes from the left ventricle of *Speg*^*+/+*^ (left panel) and *Speg*^*−/−*^ (right panel) hearts at postnatal day 1. The heart tissue was co-stained with 4′,6-diamidino-2-phenylindole (DAPI, blue) to identify nuclei. The scale bar, 10 μm. (**b**) Electron microscopy imaging of myocardium from *Speg*^*+/+*^ (left panel) and *Speg*^*−/−*^ (right panel) hearts at 18.5 dpc. Arrows point to myofibrils. N, nucleus; M, mitochondria. The scale bar, 1 μm. (**c**) Volume fraction of myofibrils, mitochondria, nuclei and undifferentiated cytoplasm in cardiomyocytes of *Speg*^*+/+*^ (white bars) and Speg^−/−^ (black bars) hearts (*n*=4 per group) was performed on electron microscopy images. Data are presented as mean±s.e.m. * versus *Speg*^*+/+*^. *P*=0.007 for myofibrils and *P*=0.004 for undifferentiated cytoplasm, using Student's unpaired *t*-test. NS, not significant.

Hearts harvested from *Speg*^*+/+*^ and *Speg*^*−/−*^ mice on postnatal day 1 were sectioned and stained for cardiac troponin T (cTnT), and confocal imaging was performed. In comparison with the mature appearing, striated cardiomyocytes of the *Speg*^*+/+*^ hearts (Fig. 1a, left panel), cardiomyocytes from *Speg*^*−/−*^ hearts revealed a lack of striations and a less organized appearance (Fig. 1a, right panel). To further assess ultrastructural changes, we harvested *Speg* mutant and wild-type hearts at 18.5 dpc and performed transmission electron microscopy. Figure 1b (left panel) demonstrated mature appearing myocytes in hearts of *Speg*^*+/+*^ mice. The myofibrils (arrows) were thick with well defined sarcomeres, and the myofibrils occupied a large portion of the cellular cytoplasm. In contrast, the myofibrils in *Speg*^*−/−*^ hearts were much thinner and loosely arranged (Fig. 1b, right panel, arrows), and the cytoplasm appears much less dense. Quantitation of the composition of cardiomyocytes by electron microscopy in the two groups revealed a marked reduction in the volume fraction of myofibrils, and an increased volume fraction of undifferentiated cytoplasm, in *Speg*^*−/−*^ compared with *Speg*^*+/+*^ cardiomyocytes (Fig. 1c). There was no significant alteration in the volume composition of mitochondria or nuclei in cardiomyocytes between the two groups. These data are consistent with immature myocytes in the *Speg*^*−/−*^ hearts, and led us to investigate CPCs in *Speg* mutant compared with wild-type hearts.

### Characterization of mouse CPCs

**Figure 2:**
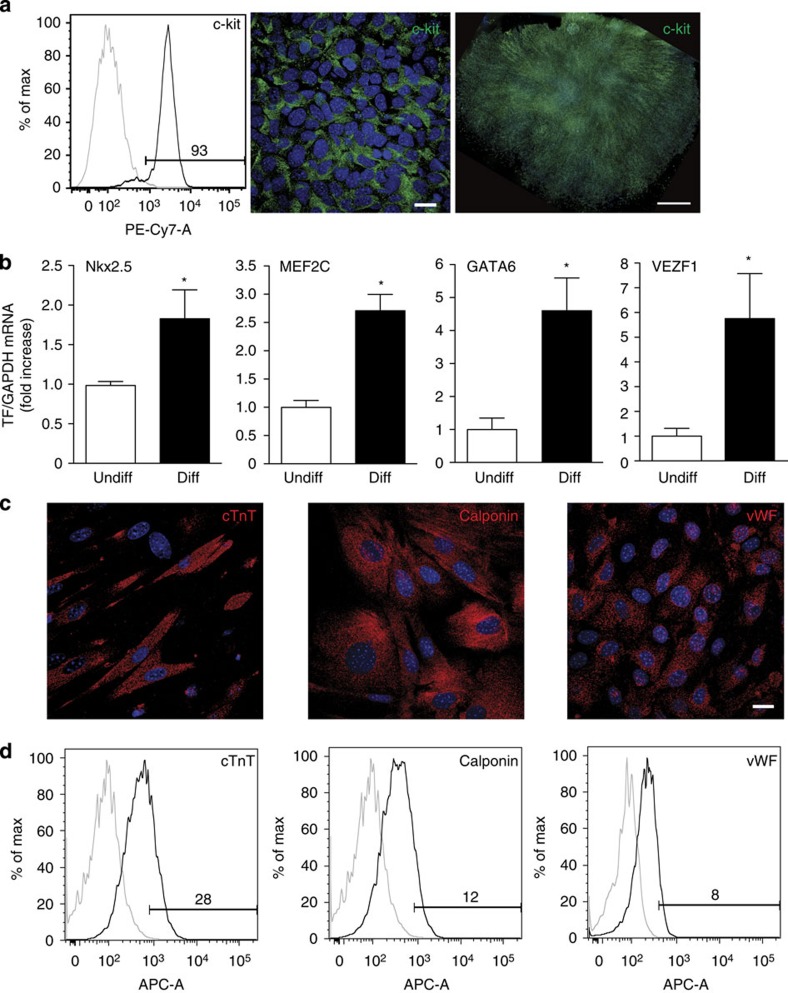
Characterization of CPCs. (**a**) Representative flow cytometric analyses assessing the percentage of c-kit-positive cells (93%) after isolation using c-kit-beads (left panel). The middle panel demonstrates confocal imaging of putative CPCs stained for c-kit (green) and DAPI (blue). The scale bar, 20 μm. In the right panel, a putative CPC clone is stained for c-kit (green) and DAPI (blue). The scale bar, 1,000 μm. (**b**) Total RNA was isolated from putative CPCs cultured in growth (Undiff) or cell-specific differentiation (Diff) medium (see methods), and quantitative real-time PCR was performed for transcription factors (TF) Nkx2.5 (*n*=5) and MEF-2C (*n*=4) for cardiomyocytes, GATA6 (*n*=4–5) for smooth muscle cells and VEZF1 (*n*=4) for endothelial cells. Data are presented as mean±s.e.m. * versus undifferentiated cells, *P*=0.026 for Nkx2.5, *P*=0.0016 for MEF-2C, *P*=0.012 for GATA6 and *P*=0.026 for VEZF1 using Student's unpaired *t*-test. (**c**) Putative CPCs were cultured in cell-specific differentiation medium (see methods) and confocal imaging of cells was performed after staining for cardiac troponin T (cTnT, red, left panel), smooth muscle calponin (red, middle panel) and von Willebrand factor (vWF, red, right panel). The scale bar, 20 μm. (**d**) Putative CPCs were cultured in medium containing 10 nM dexamethasone for unbiased differentiation, and assessed by flow cytometry for the percentage of cells positive for cTnT (28%, left panel), calponin (12%, middle panel) and vWF (8%, right panel). The black line represents the target antibody, and the grey line represents the isotype control antibody.

Hearts were harvested from either *Speg*^*+/+*^ or *Speg*^*−/−*^ mice on day 1 after birth, the tissue was digested, single-cell suspensions generated and c-kit-positive cells were isolated using fluorescence-activated cell sorting (FACS) or magnetic bead immunoselection. Initial evaluation of *Speg*^*+/+*^ cells after expansion in culture showed that 93% of cells were positive for c-kit after FACS or bead isolation (Fig. 2a, left panel). Confirmation of c-kit-positive cells is shown by confocal imaging with immunostaining for c-kit (Fig. 2a, middle panel), and fluorescent microscopy for immunostaining of c-kit in an entire clone (Fig. 2a, right panel, and a second clone in Supplementary Fig. 1) that originated from a single cell. The c-kit-positive cells harvested from *Speg*^*+/+*^ hearts showed expression of Speg by quantitative real-time PCR (qRT-PCR), but no expression was found in cells harvested from *Speg*^*−/−*^ hearts (Supplementary Fig. 2).

Cells positive for c-kit expanded in culture demonstrated a low expression of the haematopoietic marker CD34, and these cells were negative for CD133 and for markers of bone marrow-derived immune cells such as CD45, CD11b and CD11c (Supplementary Fig. 3a). We recently showed that c-kit-positive cells of embryonic, foetal and neonatal hearts are from cardiac mesoderm, and not of bone marrow origin^27^. A high percentage of c-kit-isolated cells expressed Sca1, and a subpopulation of cells showed evidence for markers CD105, CD73 and CD90.2 (Supplementary Fig. 3b). To confirm we were not isolating mesenchymal stromal cells (MSCs) from the hearts, we took CPCs and MSCs^28^ (positive control) and placed them in adipogenic differentiation medium in culture. As expected, the MSCs differentiated into adipocytes (left panel, red stain for Oil Red O), however *Speg*^*+/+*^ (middle panel) and *Speg*^*−/−*^ (right panel) CPCs showed no evidence of adipocyte differentiation (Supplementary Fig. 4). These data confirm that our cells-of-interest were not MSCs harvested from newborn hearts. In addition, the relationship between c-kit-positive CPCs and the MSC hierarchy of cells remains unknown.

The c-kit-isolated cells from both *Speg*^*+/+*^ and *Speg*^*−/−*^ hearts were predominantly negative for a marker of commitment (GATA4) and also for transcription factors and cytoplasmic proteins characteristic of cardiomyocytes (Nkx2.5 and sarcomeric α-actin, respectively), smooth muscle cells (GATA6 and calponin) and endothelial cells (Ets 1, CD31 and Flk-1) (Supplementary Fig. 3c). These data support the fact that the c-kit-isolated cells were undifferentiated, and there was no apparent difference between *Speg*^*+/+*^ and *Speg*^*−/−*^ cells. When *Speg*^*+/+*^ cells were placed in specialized differentiation media for different cell types in culture, qRT-PCR revealed increased expression of transcription factors for commitment into cardiomyocyte (Nkx2.5 and MEF-2C), smooth muscle cell (GATA6) and endothelial (VEZF1) lineages compared with non-differentiated cells (Fig. 2b). In these same differentiation conditions, staining for immuocytochemistry demonstrated that c-kit-isolated cells from *Speg*^*+/+*^ hearts differentiated into cardiomyocytes, by staining for cTnT (Fig. 2c, left panel); smooth muscle cells, by staining for calponin (Fig. 2c, middle panel); and endothelial cells, by staining for von Willebrand factor (vWF, Fig. 2c, right panel). When the CPCs were cultured in medium containing dexamethasone, a nonselective stimulus for CPC differentiation (but not a potent stimulus for any specific lineage), flow cytometry demonstrated that the c-kit-isolated cells from *Speg*^*+/+*^ hearts also differentiated into cardiomyocytes (cTnT, Fig. 2d, left panel), smooth muscle cells (calponin, Fig. 2d, middle panel) and endothelial cells (vWF, Fig. 2d, right panel). These data reveal that *Speg*^*+/+*^ CPC are capable of differentiating into three different cell types; and when placed in a nonselective differentiation medium, the CPCs have a tendency to differentiate into cardiomyocytes (28%) more than smooth muscle cells (12%) or endothelial cells (8%) (Fig. 2d). Furthermore, the expression level of Speg mRNA increased threefold in wild-type CPCs placed in cardiomyocyte differentiation conditions in culture (Supplementary Fig. 2).

### Properties of CPCs harvested from *Speg* ^*+/+*^ and *Speg* ^*−/−*^ mice

**Figure 3:**
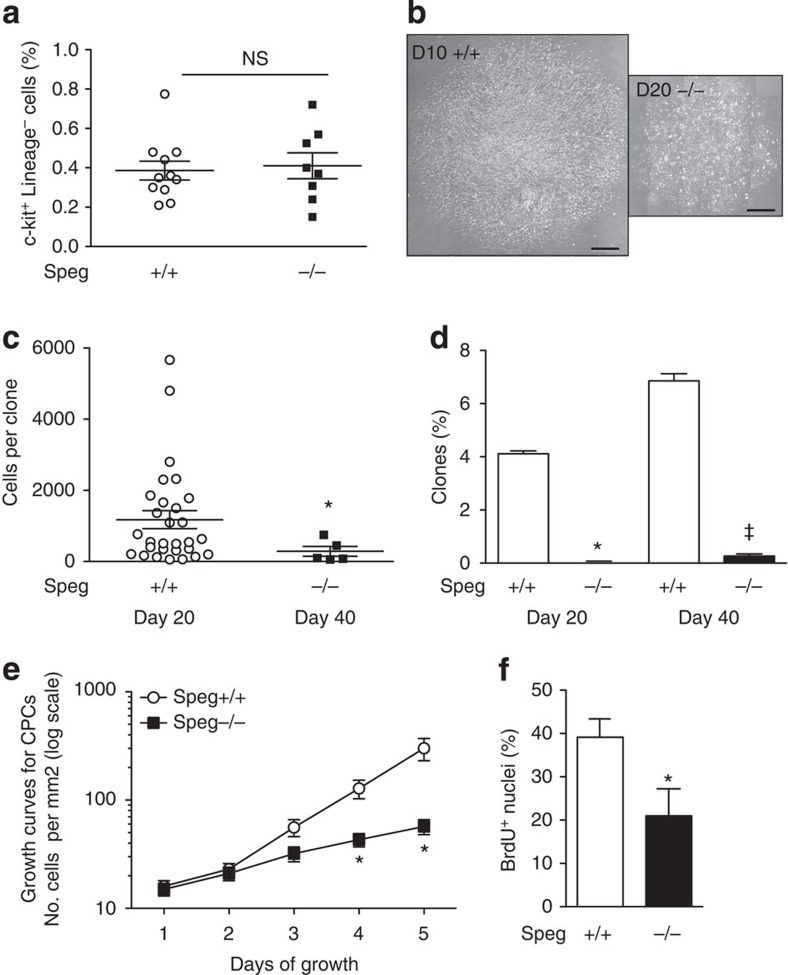
Clone formation and growth curves of CPCs. (**a**) Percentage of c-kit-positive (+) Lineage-negative (−) cells from hearts of *Speg*^*+/+*^ (white circles, *n*=11) and *Speg*^*−/−*^ (black squares, *n*=8) mice by flow cytometry. Hearts from day 1 pups were harvested and total cells were collected following enzyme digestion. NS, not significant using Student's unpaired *t*-test. (**b**) CPCs were plated in limited dilution, and monitored in growth medium. Phase contrast images of representative clones of *Speg*^*+/+*^ CPCs after 10 days in culture (D10+/+, left panel) and *Speg*^*−/−*^ CPCs after 20 days in culture (D20−/−, right panel). The scale bar, 250 μm. (**c**) Quantitation of cells per clone after 20 days in culture for *Speg*^*+/+*^ clones (white circles, *n*=29), and after 40 days in culture for *Speg*^*−/−*^ clones (black squares, *n*=5). *P<0.01 versus *Speg*^*+/+*^ clones using Mann–Whitney *U*-test. (**d**) *Speg*^*+/+*^ (white bars) and *Speg*^*−/−*^ (black bars) CPCs were assessed for efficiency of clone formation after limited dilution plating, and depicted as a percentage of total cells forming clones. *P<0.01 versus *Speg*^*+/+*^ at Day 20, and ^‡^*P*<0.001 versus *Speg*^*+/+*^ at Day 40 using one-way analysis of variance, followed by Newman–Keuls multiple comparison test. These experiments were performed a minimum of three independent times. (**e**) *Speg*^*+/+*^ (white circles, *n*=8) and *Speg*^*−/−*^ (black squares, *n*=4) CPCs were plated, and the number of cells per mm^2^ was assessed daily over a 5-day period. *P=0.007 at day 4, and *P*=0.0032 at day 5 versus *Speg*^*+/+*^ cells using two-way analysis of variance. (**f**) *Speg*^*+/+*^ (white bar, *n*=10) and *Speg*^*−/−*^ (black bar, *n*=6) CPCs were plated and grown under growth conditions, labelled with BrdU and the percentage of nuclei incorporating BrdU was assessed at day 5. *P=0.031 versus *Speg*^*+/+*^ cells using Student's unpaired *t*-test. For **a**,**c**–**f** data are presented as mean±s.e.m.

Initially we investigated whether there was a disparity in the number of c-kit-positive, lineage-negative cells in *Speg* mutant compared with wild-type hearts at day 1 after birth. As shown in Fig. 3a, there was no difference in the percentage of c-kit-positive, lineage-negative cells (expressed as a percentage of total cells) harvested from *Speg*^*+/+*^ (0.39±0.05) and *Speg*^*−/−*^ (0.41±0.07) hearts. We next assessed whether a decrease in the expression of Speg would have an effect on CPC function *in vitro*, by investigating progenitor cell properties (clone formation, self-renewal and multipotency) of c-kit-isolated CPCs in each group. These studies were performed using limited dilution assays, as *Speg*^*−/−*^ cells did not survive after FACS when seeded in Terasaki plates. Clones formed from *Speg* mutant CPCs were much smaller than clones from wild-type CPCs, even when cultured for longer periods of time (Fig. 3b). Moreover, quantitation of the number of cells per clone was significantly less in *Speg*^*−/−*^ CPCs (286±137) at day 40 compared with *Speg*^*+/+*^ CPCs (1,176±254) at day 20 (Fig. 3c). Clonal efficiency was assessed, and the percentage of clones formed from *Speg*^*−/−*^ CPCs was dramatically decreased compared with clonal efficiency of wild-type CPCs (Fig. 3d). In fact, very few clones were detected from *Speg* mutant CPCs until 40 days after plating (0.27±0.03%) compared with wild-type CPCs (6.84±0.47%). Proliferation of *Speg* mutant and wild-type CPCs were next assessed *in vitro*. An identical number of cells were plated, and then counted daily over a 5-day period. Proliferation of *Speg*^*−/−*^ CPCs (black squares) was significantly less than *Speg*^*+/+*^ cells (white circles) by days 4 and 5 (Fig. 3e). We analysed the logarithmic growth of the cells, and the rate was 2.6±0.5-fold greater for the *Speg*^*+/+*^ compared with the *Speg*^*−/−*^ cells. This difference in growth of cultured cells was also confirmed by bromodeoxyuridine (BrdU) labelling on day 5 (Fig. 3f), with the rate of growth 1.9±0.6 fold greater for *Speg*^*+/+*^ compared with the *Speg*^*−/−*^ cells. These data demonstrate the growth potential was significantly reduced in *Speg* mutant versus wild-type CPCs.

**Figure 4:**
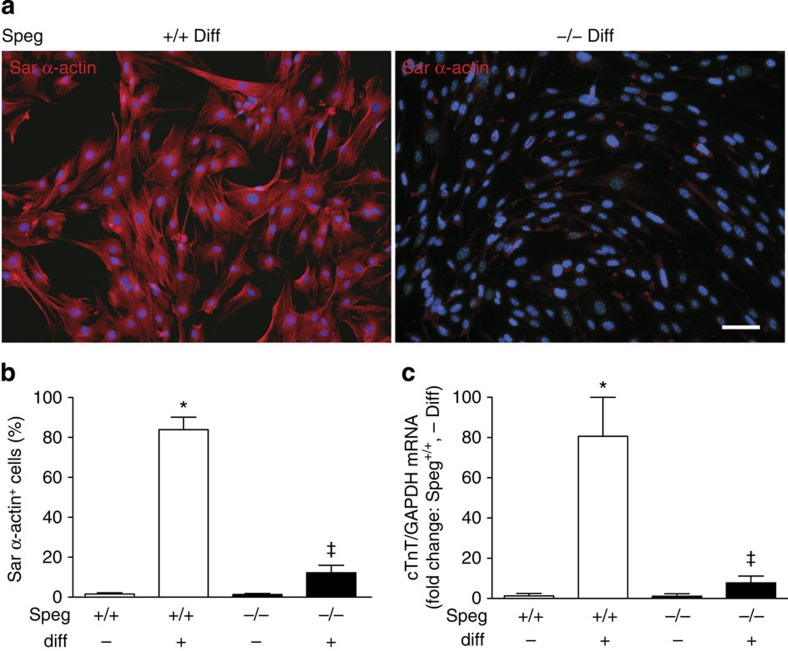
Differentiation of *Speg*^*+/+*^ and *Speg*^*−/−*^ CPCs into cardiomyocytes. (**a**) CPCs were cultured in cardiomyocyte differentiation (+Diff) or growth (−Diff) medium (see Methods). Representative images of immunofluorescence staining for sarcomeric (Sar) α-actin (red) in *Speg*^*+/+*^ (left panel) and *Speg*^*−/−*^ (right panel) cells after 7 days of differentiation. The scale bar, 100 μm. (**b**) Immunofluorescence staining for Sar α-actin was quantitated in *Speg*^*+/+*^ (white bars) and *Speg*^*−/−*^ (black bars) CPCs after culture in medium to retain the cells in an undifferentiated state (−) or in medium to promote cardiomyocyte differentiation (+). Data are presented as mean±s.e.m. *P<0.0001, *Speg*^*+/+*^ +Diff (*n*=6) versus *Speg*^*+/+*^ −Diff (*n*=5). ^‡^*P*<0.0001, *Speg*^*−/−*^ +Diff (*n*=5) versus *Speg*^*+/+*^ +Diff (*n*=6) using one-way analysis of variance, followed by Newman–Keuls multiple comparison test. (**c**) qRT-PCR was performed for cardiac troponin T (cTnT) on RNA extracts from *Speg*^*+/+*^ (white bars) and *Speg*^*−/−*^ (black bars) CPCs after culture in medium to retain the cells in an undifferentiated state (−Diff), or in medium to promote cardiomyocyte differentiation (+Diff). Expression levels of cTnT mRNA were divided by expression levels of the control gene GAPDH, and shown as a fold increase in expression versus *Speg*^*+/+*^ −Diff. Data are presented as mean±s.e.m. *P=0.015, *Speg*^*+/+*^ +Diff (*n*=3) versus *Speg*^*+/+*^ −Diff (*n*=3). ^‡^*P*=0.007, *Speg*^*−/−*^ +Diff (*n*=4) versus *Speg*^*+/+*^ +Diff (*n*=3) using one-way analysis of variance, followed by Newman–Keuls multiple comparison test.

In the final assessment of progenitor cell properties, we assessed the differentiation of *Speg*^*+/+*^ and *Speg*^*−/−*^ CPCs into cardiomyocytes *in vitro*. The majority of wild-type CPCs (83.8±6.2%) were able to differentiate into cardiomyocytes using specialized differentiation medium in culture, as confirmed by immunocytochemical staining for sarcomeric α-actin (Fig. 4a, left panel). In contrast, markedly fewer CPCs from *Speg* mutant hearts (11.9±3.8%) were able to differentiate into cardiomyocytes (Fig. 4a, right panel). Figure 4b shows the quantitative analysis of the immunostaining for sarcomeric α-actin in *Speg*^*+/+*^ and *Speg*^*−/−*^ CPCs, in the presence or absence of differentiation medium. This defect in differentiation of *Speg* mutant CPCs was also confirmed at the level of mRNA by performing qRT-PCR for cTnT in the cells, in the presence or absence of differentiation medium (Fig. 4c). In both of these analyses, the expression levels for markers of cardiomyocytes were markedly lower in *Speg*^*−/−*^ CPCs compared with Speg^+/+^ CPCs exposed to differentiation medium. Taken together, our data demonstrate that CPCs harvested from *Speg* mutant hearts have a decreased ability to form clones, to proliferate and to differentiate into cardiomyocytes compared with wild-type CPCs.

### Rescue of *Speg* ^*−/−*^ mice by *in utero* injection of *Speg* ^*+/+*^ CPCs

**Figure 5:**
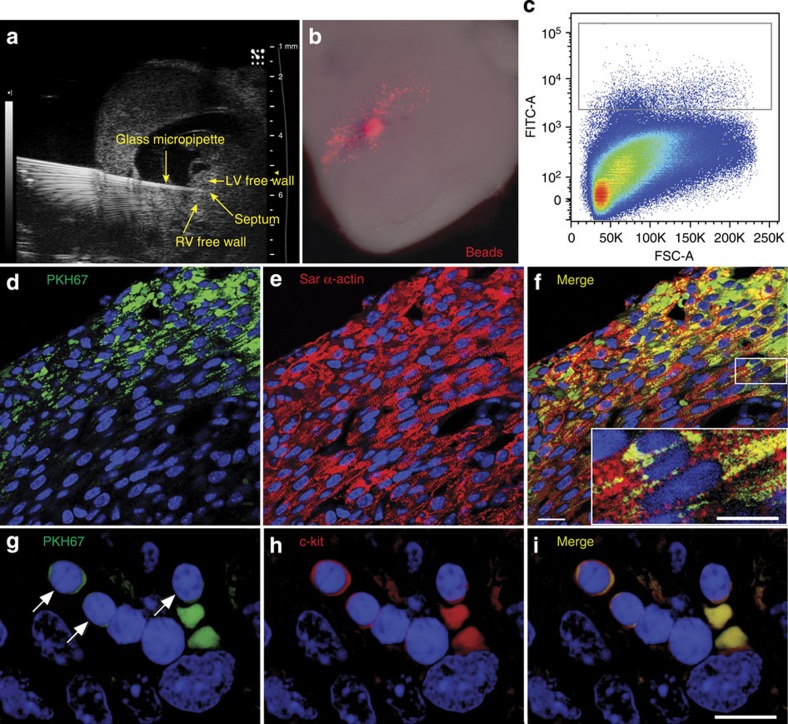
Engraftment of cells in recipient hearts after *in utero* injection of CPCs. (**a**) CPCs were injected into the myocardium of a wild-type mouse with a beveled glass micropipette, under echocardiographic guidance, at 13.5 dpc. (**b**) The CPCs were co-injected with rhodamine-conjugated beads (red), to demonstrate the instillation of cells. (**c**) On day 1 after birth (19.5 dpc), hearts injected with CPCs (labelled with PKH67, green) were digested, the cells dissociated and flow cytometry was performed to identify the presence of exogenously administered cells (FITC for PKH67 in the pseudo-colour density plot). The positive cells are demarcated in the rectangular gated area. (**d**–**f**) Representative images of heart sections day 1 after birth, showing immunofluorescence for PKH67-labelled exogenous cells (**d**, green), sarcomeric (Sar) α-actin-positive cardiomyocytes (**e**, red), and merged images showing double positive cells (**f**, yellow). The sections were also counter stained with DAPI (blue). The scale bars, 20 μm (**d**–**f**); 10 μm (**f** inset). Double positive cells (from the region outlined by the small rectangle) are shown in a higher magnification inset of the larger rectangle (**f**, yellow). (**g**–**i**) Representative images of heart sections day 1 after birth, showing a cluster of exogenous cells with immunofluorescence for PKH67 (**g**, green with arrows), c-kit-positive (**h**, red) and merged images showing double positive cells (**i**, yellow). The two fluorescent cells with an absence of nuclei (no DAPI staining, **g**–**i**) are red blood cells. The scale bar, 10 μm (**g**–**i**).

Under echocardiographic guidance, using a beveled glass micropipette, we initially injected CPCs—expanded in culture and then fluorescently labelled green (PKH67)—into hearts of wild-type mouse foetuses at 13.5 dpc (Fig. 5a, example of injection into the septum). Figure 5b shows the location of injected cells (septum example) by co-injecting with rhodamine-labelled beads. For the rescue experiments, injections were given into the walls of the left ventricle (LV), septum and right ventricle (RV) (see Methods for details). After injection, the pups were allowed to be born naturally 6 days later, and the hearts were harvested on day 1 after birth (19.5 dpc). In a subgroup of mice, the hearts were digested, the cells dissociated and flow cytometry performed. Figure 5c displays a pseudo-colour density plot, with evidence for exogenously administered CPCs in day 1 hearts (demarcated in the rectangular gated area). The confocal images show green fluorescence for the exogenously injected CPCs (Fig. 5d), and red staining for sarcomeric α-actin, highlighting cardiomyocytes (Fig. 5e). In the merged image (Fig. 5f), there is evidence for engrafted CPCs that subsequently differentiated into cardiomyocytes (yellow), and also CPCs that remained in an undifferentiated state (green). Figure 5f (inset) shows a higher power view of a merged image. The CPCs that have differentiated into cardiomyocytes (yellow) have an appearance very similar to endogenous myocytes (red). In addition, c-kit-positive CPCs harvested from transgenic mice expressing enhanced green fluorescent protein (GFP) under the direction of the human ubiqutin C promoter showed this same mature appearance (Supplementary Fig. 5). To further assess undifferentiated CPCs in the myocardium, confocal microscopy was performed. We found a cluster of exogenous CPCs (green, Fig. 5g), which also stained positive for c-kit (red, Fig. 5h). The merged image (Fig. 5i) reveals a cluster of undifferentiated, exogenous CPCs (yellow), forming a putative niche in the myocardium. Taken together, these data demonstrate that not only do CPCs injected *in utero* differentiate into mature appearing cardiomyoctyes postnatally, but a subpopulation of CPCs remains in an undifferentiated state and form putative niches. Thus, we next targeted injection of foetuses from *Speg*^*+/−*^ breeding.

**Figure 6:**
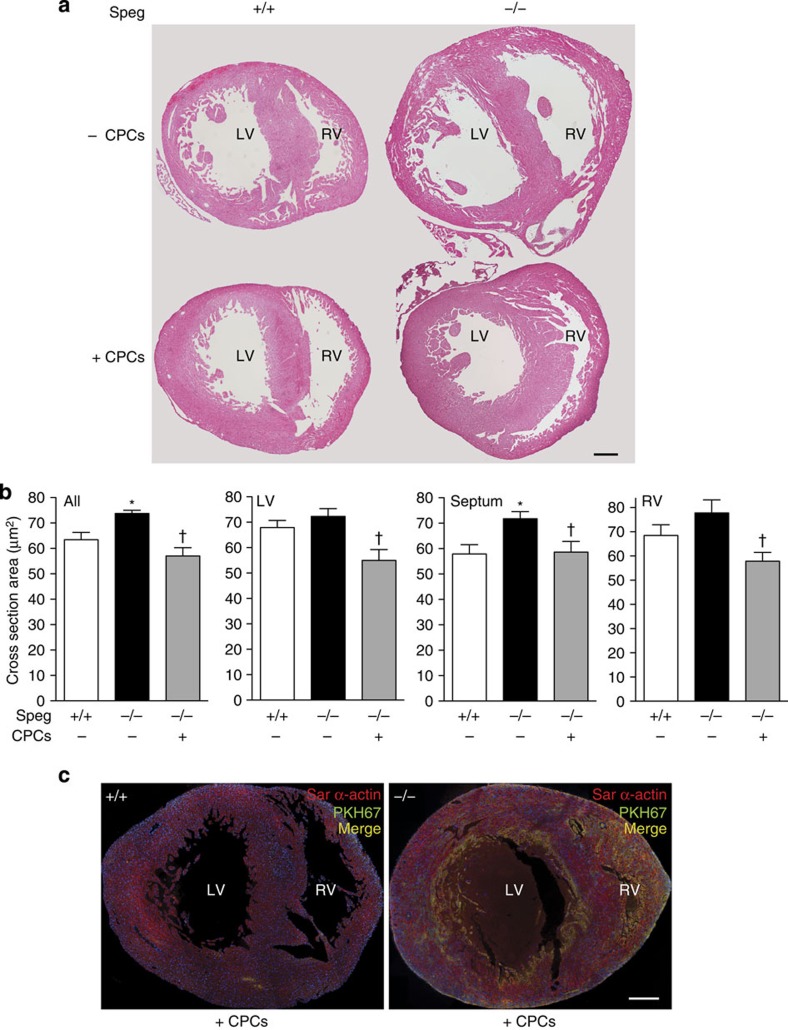
Regeneration of myocardium in *Speg*^*−/−*^ hearts after *in utero* injections of CPCs. (**a**) *Speg*^*+/+*^ and *Speg*^*−/−*^ mice were administered intra-cardiac injections of vehicle (−CPCs) or CPCs (+CPCs) with a beveled glass micropipette, under echocardiographic guidance, at 13.5 dpc. The hearts were harvested on day 1 after birth (19.5 dpc) for histological analyses. Representative images of haematoxylin and eosin-stained sections, from a minimum of five hearts per group, are shown for *Speg*^*+/+*^ and *Speg*^*−/−*^ hearts. (**b**) Cross-section area (μm^2^) of cardiomyocytes was analysed in hearts from *Speg*^*+/+*^ and *Speg*^*−/−*^ mice not receiving CPCs (white and black bars, respectively, *n*=5 per group), and *Speg*^*−/−*^ mice injected with wild-type CPCs (grey bars, *n*=7). Cardiomyocytes were assessed from the left ventricle (LV), septum and right ventricle (RV), and also a composite of cells from all three regions. About 100 cells were measured per region of myocardium, from each mouse. Cross-section area (μm^2^) is presented as mean±s.e.m. *P*=0.003, * versus *Speg*^*+/+*^ cardiomyocytes no (−) CPCs, † versus *Speg*^*−/−*^ cardiomyocytes no (−) CPCs using one-way analysis of variance, followed by Newman–Keuls multiple comparison test. (**c**) *Speg*^*+/+*^ and *Speg*^*−/−*^ mice were administered intra-cardiac injections of PKH67 (green) labelled CPCs under echocardiographic guidance, at 13.5 dpc. The hearts were harvested on day 1 after birth (19.5 dpc) for histological analyses. Immunofluorescent staining was performed for sarcomeric (Sar) α-actin (red) and DAPI (blue). Merged confocal images show CPCs that have engrafted and differentiated into cardiomyocytes (yellow). In both **a**,**c**, the scale bar, 200 μm. LV, left ventricle; RV, right ventricle.

Figure 6a shows lower power views of *Speg*^*+/+*^ and *Speg*^*−/−*^ hearts (day 1 after birth) that received injections of CPCs (+) after expansion in culture, or no CPCs (−), at 13.5 dpc. The *Speg* mutant hearts that received no CPCs appear larger than wild-type hearts, with no evidence for thickening of the right or left ventricular walls (Fig. 6a, right upper panel compared with left upper panel). While administration of CPCs had no apparent effect on *Speg*^*+/+*^ hearts (left lower panel), the *Speg*^*−/−*^ hearts receiving CPCs (right lower panel) appeared smaller than *Speg*^*−/−*^ hearts receiving no CPCs. We also assessed the hearts at the level of cardiomyocyte size (Fig. 6b). Overall cardiomyocyte cross-sectional area was larger in *Speg*^*−/−*^ compared with *Speg*^*+/+*^ hearts, consistent with hypertrophic cells, and this was demonstrated most prominently in myocytes sampled from the septum. However, after injection of wild-type CPCs into *Speg*^*−/−*^ hearts, the myocytes from the LV, septum and RV all demonstrated a reduction in cross-sectional area comparable to the size of cardiomyocytes from *Speg*^*+/+*^ hearts (Fig. 6b). Additional staining for sarcomeric α-actin (red) was performed on *Speg*^*+/+*^ and *Speg*^*−/−*^ hearts on day 1 after birth (Fig. 6c). These mice received intra-cardiac injections of green fluorescently labelled CPCs on 13.5 dpc. Engrafted CPCs that differentiated into cardiomyocytes are demonstrated by yellow staining (Fig. 6c, merged images). Engraftment and differentiation of CPCs appeared throughout the RV wall and more localized in the subendocardial regions of the LV and septum in the *Speg*^*−/−*^ heart, compared with a diffuse appearance in the *Speg*^*+/+*^ heart. However, analysis of entire hearts from all *Speg* genotypes revealed no differences in the magnitude of CPC engraftment and differentiation (see Supplementary Fig. 7). Please note there is a blood clot in the chamber of the LV of the *Speg*^*−/−*^ heart in Fig. 6c.

**Figure 7:**
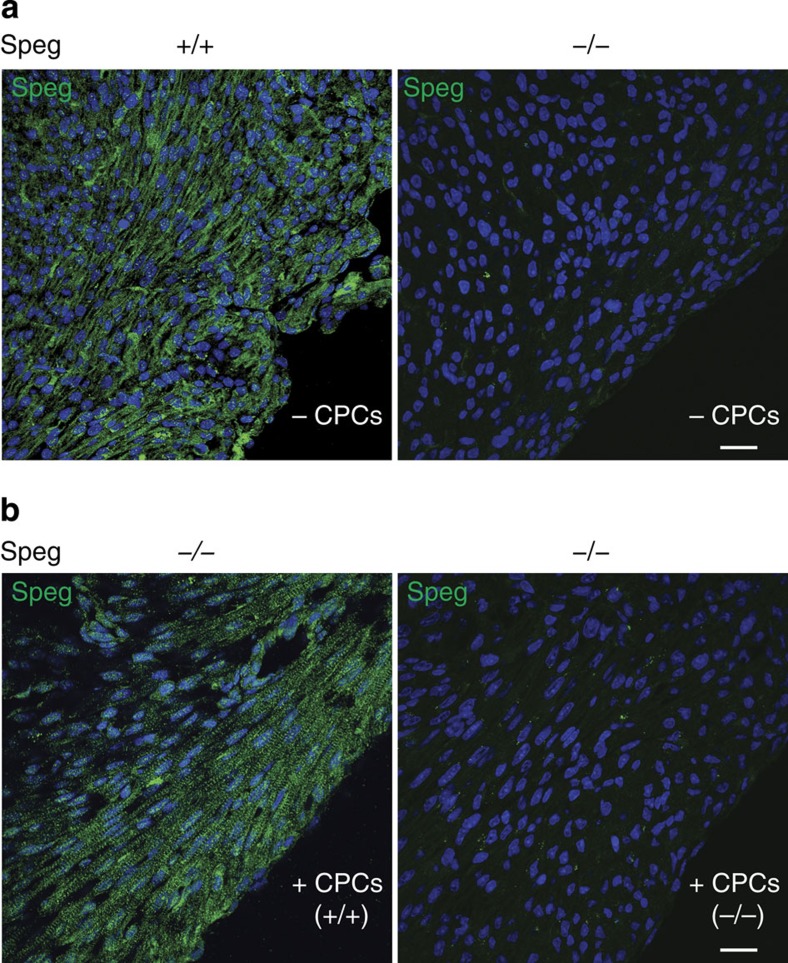
Expression of Speg in mutant hearts after *in utero* injections of CPCs. Confocal imaging of Speg immunostaining (green) in (**a**) *Speg*^*+/+*^ heart (left panel) and in *Speg*^*−/−*^ heart (right panel) receiving no CPCs (−CPCs); and (**b**) representative images of *Speg*^*−/−*^ hearts receiving wild-type CPCs (+CPCs+/+, left panel) or mutant CPCs (+CPCs−/−, right panel). In total, immunostaining for Speg was performed on hearts of 13 *Speg*^*−/−*^ pups (from 9 dams) receiving wild-type CPCs, and 3 *Speg*^*−/−*^ pups (from 6 dams) receiving mutant CPCs. The sections in **a**,**b** were also counter stained with DAPI (blue), and the scale bars, 20 μm.

To further assess the expression of Speg, in the presence or absence of *in utero* injections of cultured CPCs, we performed immunostaining for Speg in day 1 postnatal hearts. Fluorescent microscopy images revealed Speg staining of cardiomyocytes in wild-type (+/+) hearts not receiving CPCs (−) (Fig. 7a, left panel), but no evidence of immunostaining for Speg in mutant hearts (−/−) not receiving CPCs (Fig. 7a, right panel). However, after injection of +/+CPCs into *Speg*^*−/−*^ hearts at 13.5 dpc, the hearts of day 1 neonates revealed regions of immunostaining for Speg (Fig. 7b, left panel), which was not present in *Speg*^*−/−*^ hearts receiving −/−CPCs (Fig. 7b, right panel).

**Figure 8:**
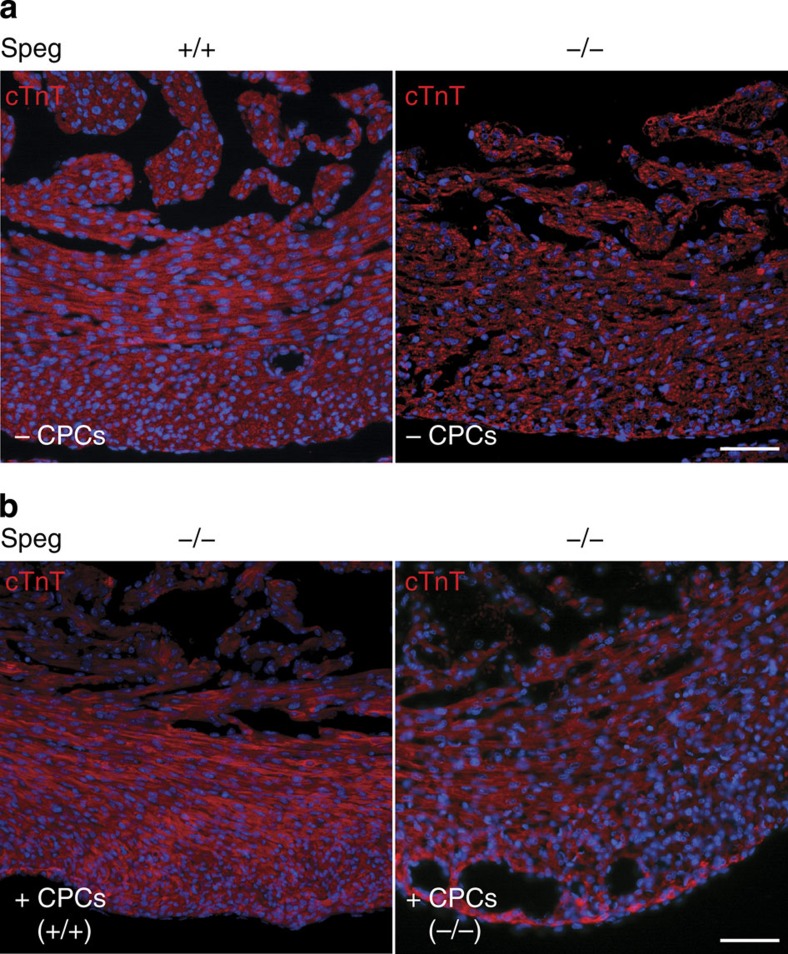
Maturation of cardiomyocytes in *Speg*^*−/−*^ **hearts after** ***in utero*** **injections of CPCs.** Confocal imaging of cardiac troponin T (cTnT, red) in (**a**) *Speg*^***+/+***^ heart (left panel) and *Speg*^*−/−*^ heart (right panel) receiving no CPCs (−CPCs), and (**b**) representative images of *Speg*^*−/−*^ hearts receiving wild-type CPCs (+CPCs +/+, left panel) and *Speg*^*−/−*^CPCs (+CPCs −/−, right panel). Hearts for cTnT immunostaining were performed on hearts of 13 *Speg*^*−/−*^ pups (from 9 dams) receiving wild-type CPCs, and 3 *Speg*^*−/−*^ pups (from 6 dams) receiving mutant CPCs, as described in Fig. 7b. The sections in **a**,**b** were also counter stained with DAPI (blue), and the scale bars, 50 μm.

The appearance of cardiomyocytes in the ventricular wall showed that analogous to Fig. 1a, *Speg*^*+/+*^ hearts have well-organized cardiomyocytes (Fig. 8a, left panel), while *Speg*^*−/−*^ hearts have a disorganized appearance by fluorescence microscopy (Fig. 8a, right panel). However, injection of cultured wild-type CPCs into *Speg*^*−/−*^ hearts revealed a much more organized appearance of the mutant myocardium at the cellular level, with evidence of well-organized myofibres (Fig. 8b, left panel). Interestingly, *Speg*^*−/−*^ hearts injected with cultured *Speg* mutant CPCs (Fig. 8b, right panel) showed some improvement compared with *Speg*^*−/−*^ hearts not receiving CPCs, however, the structural appearance of these hearts was more disorganized, with inconsistent myofibre alignment by cTnT immunostaining compared with *Speg*^*−/−*^ hearts receiving wild-type CPCs (Fig. 8b, left panel).

**Figure 9:**
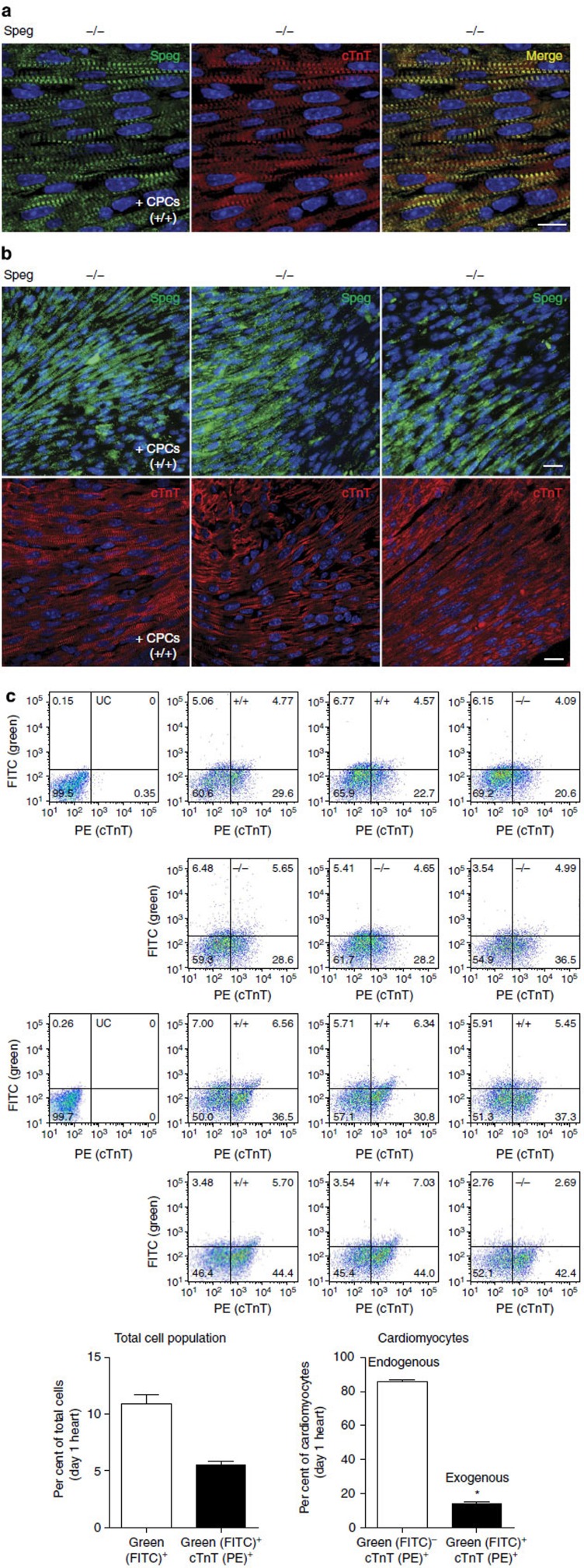
Wild-type CPCs engraft and promote maturation of *Speg*^*−/−*^ hearts. (**a**) Confocal imaging of immunostaining for Speg (green, left panel), cTnT (red, middle, panel) and merged image (yellow, right panel) in *Speg*^*−/−*^ heart receiving wild-type CPCs (+CPCs +/+) injections. The scale bar, 10 μm. (**b**) Representative confocal imaging of immunostaining for Speg (green, upper panels) in three individual *Speg*^*−/−*^ hearts receiving wild-type CPCs (+CPCs +/+). In the lower panels, confocal imaging of immunostaining for cardiac troponin T (cTnT, red) in the same *Speg*^*−/−*^ hearts receiving wild-type CPCs (+CPCs +/+). Overall, immunostaining for Speg was performed on hearts of 13 *Speg*^*−/−*^ pups (from 9 dams) receiving wild-type CPCs. The scale bar, 20 μm. (**c**) Upper panels show 12 representative flow cytometry pseudo-colour density plots (from a total of 20 neonatal hearts, of 3 pregnant dams) from cells harvested from wild-type (+/+) and *Speg* mutant (−/−) hearts at day 1, assessing green fluorescently labelled cells (FITC) and cells expressing cTnT (PE). Unstained cells (UC) serve as a negative control, and are shown in two flow cytometry pseudo-colour density plots on the left side of the upper panel. The left lower panel demonstrates the percentage of total cells that are green (white bar, *n*=20), or green cells expressing cTnT (black bar, *n*=20). The right lower panel demonstrates percentage of cTnT-positive cardiomyocytes that are not green (endogenous, white bar, *n*=20) or green (exogenous, black bar, *n*=20). *P*<0.0001; * versus endogenous cardiomyocytes using Student's unpaired *t*-test.

To determine whether the engrafted wild-type CPCs in the *Speg*^*−/−*^ hearts differentiated into mature cardiomyocytes, *Speg*^*−/−*^ hearts were co-immunostained for both Speg and cTnT. As seen by confocal microscopy in Fig. 9a, the Speg-expressing cells (green, left panel) in the mutant heart also expressed cTnT (red, middle panel), with colocalization between Speg and cTnT (yellow, right panel). Thus, engrafted wild-type CPCs are capable of differentiating into mature, striated cardiomyocytes in *Speg*^*−/−*^ hearts. Speg immunostaining was present in localized regions of all *Speg*^*−/−*^ hearts injected with wild-type CPCs (Fig. 9b, upper panels, three hearts from different mice), however, the presence of striated, mature and well-organized cardiomyocytes (by confocal imaging of cTnT immunostaining) was evident throughout the myocardium of these *Speg*^*−/−*^ hearts, even outside of the regions of CPC engraftment (Fig. 9b, lower panels). To further explore this concept, we repeated injections of wild-type CPCs into foetuses at 13.5 dpc, and on day 1 after birth the hearts were harvested, enzymatically digested into single-cell suspensions and flow cytometry was performed to assess green fluorescently labelled cells (originating from the CPCs), and also for cells staining for cTnT. Twelve representative flow cytometry pseudo-colour density plots of our analyses are shown in Fig. 9c (upper panels), along with unstained cells as a negative control. The complete gating strategy for these flow cytometry experiments is shown in Supplementary Fig. 6. Quantification of the flow cytometry data revealed that on day 1 after birth, 6 days after the injection, 10.9±0.8% of the total cell population was fluorescein isothiocyanate (FITC)-positive, fluorescently labelled cells originating from the injected CPCs (Fig. 9c, lower left panel). Moreover, 5.6±0.3% of the total cell population was FITC positive and also stained positive for cTnT. These FITC-positive, cTnT-positive cells represent CPCs that have differentiated into cardiomyocytes in the day 1 hearts, although we cannot exclude a component of cell fusion contributing to this population of cardiomyoctyes. Additional assessment of entire hearts from CPC recipients demonstrated no difference in CPC engraftment, or differentiation of these cells into cardiomyocytes, between *Speg* genotypes (Supplementary Fig. 7a). Further analyses revealed that in the hearts, 14.2±0.9% of the cardiomyocytes were derived from exogenous injected CPCs, while 85.8±0.9% were composed of endogenous (FITC negative, cTnT positive) cardiomyocytes (Fig. 9c, lower right panel). Also, these data were analogous between *Speg* genotypes (Supplementary Fig. 7b). Supplementary Table 1 provides the primary data regarding the flow cytometry analyses. Taken together, these data suggest that maturation of the *Speg*^*−/−*^ myocardium in mice receiving wild-type CPCs goes beyond the concept of just CPC engraftment and replacement of the abnormal myocardium, and that wild-type CPCs may have paracrine actions that promote the maturation of endogenous *Speg*^*−/−*^ cardiomyocytes.

Beyond maturation, we also assessed the effect of injecting CPCs expanded in culture on the number of endogenous cardiomyocytes in recipient hearts, compared with cells from hearts not injected with CPCs. As shown in Supplementary Fig. 8 (flow cytometry pseudo-colour density plots of wild-type and *Speg* mutant recipients in a; and composite data in b), hearts receiving CPCs had a very similar percentage of cardiomyocytes in their endogenous cell population (38.3±1.4%) compared with hearts of mice not injected with CPCs (39.9±2.8%). Furthermore, these percentages of cardiomyoctyes were very similar between different *Speg* genotypes, in both groups (Supplementary Fig. 8b, right panel). Supplementary Table 2 provides the primary data regarding these analyses.

**Figure 10:**
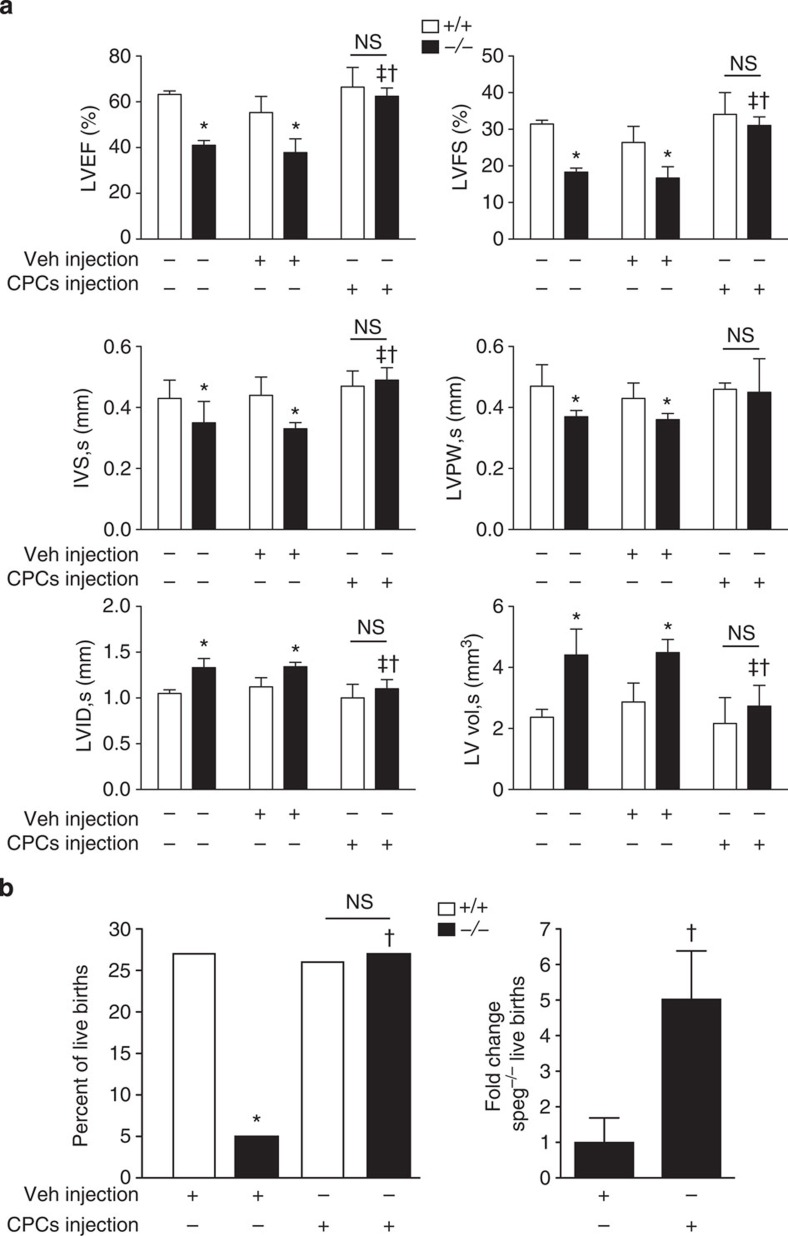
Functional assessment of *Speg*^*−/−*^ and *Speg*^*+/+*^ hearts after *in utero* injections of CPCs. (**a**) *Speg*^*+/+*^ (white bars, *n*=6–7 per group) and *Speg*^*−/−*^ (black bars, *n*=4–5 per group) mice received either no injection (−), or intra-cardiac injection (+) with vehicle (Veh) or CPCs at 13.5 dpc. On day 1 after birth (19.5 dpc), echocardiograms were performed to assess cardiac function. The left ventricles were assessed for ejection fraction (LVEF) and fractional shortening (LVFS). Measurements for thickness of the intraventricular septum (IVS) and LV posterior wall (LVPW), and dimensions of the LV internal diameter (LVID) and LV volume (LV vol) were performed during systole (s). Data are presented as mean±s.e.m. *P<0.05 versus *Speg*^*+/+*^ mice in the same group. ^‡^*P*<0.05 versus *Speg*^*−/−*^ mice receiving no injection. ^†^*P*<0.05 versus *Speg*^*−/−*^ mice receiving Vehicle injection. NS, not significant. Analyses performed using one-way analysis of variance, followed by either Bonferroni's or Newman–Keuls multiple comparison test. (**b**) Foetuses of *Speg*^*+/−*^ pregnant dams were injected with either Vehicle (Veh, *n*=6 litters) or wild-type CPCs (*n*=6 litters) at 13.5 dpc. On day 1 after birth (19.5 dpc), the per cent of live births was assessed in *Speg*^*+/+*^ (white bars) and *Speg*^*−/−*^ (black bars) pups (left panel). *P*=0.0012; * versus *Speg*^*+/+*^ pups, Vehicle injection; † versus *Speg*^*−/−*^ pups, Vehicle injection. Analysis performed by Fisher's exact test. In the right panel, fold change (mean±s.e.m.) in live births of *Speg*^*−/−*^ pups receiving either Vehicle or wild-type CPCs injection. *P*=0.0198; † versus *Speg*^*−/−*^ pups, Vehicle injection using Student's unpaired *t*-test.

Echocardiograms performed on day 1 after birth revealed that *Speg*^*−/−*^ mice (not receiving injections of vehicle or CPCs) compared with *Speg*^*+/+*^ mice had a significant decrease in LV ejection fraction (EF: 41±2.1% versus 63±1.5%) and fractional shortening (FS: 18±1.1% versus 31±1.1%), respectively (Fig. 10a). Moreover, these *Speg*^*−/−*^ mice had a significant decrease in the thickness of their septal and LV posterior walls, and an increase in their LV internal diameters and volumes (Fig. 10a). These changes were distinctly apparent during systole, and not diastole (Supplementary Fig. 9), which is consistent with an abnormality in contractile function. To determine whether *in utero* administration of exogenous wild-type CPCs into *Speg* mutant hearts would improve cardiac function, we injected CPCs expanded in culture (or vehicle without cells) into hearts at 13.5 dpc, using micro-ultrasound guidance, and echocardiograms were performed on day 1 after birth (19.5 dpc). The echocardiograms revealed that *Speg*^*−/−*^ mice receiving intra-cardiac injections at 13.5 dpc, but no CPCs, had no difference in LVEF and LVFS compared with *Speg*^*−/−*^ mice receiving no injections (Fig. 10a). Thus, the injection itself at 13.5 dpc had no effect on cardiac function postnatally. In *Speg*^*−/−*^ mice receiving wild-type CPCs, their LVEF and LVFS increased to 62±4% and 31±2%, respectively, a level of function analogous to *Speg*^*+/+*^ mice. While septal and LV posterior walls were thinner in *Speg*^*−/−*^ mice, mutant mice receiving CPCs had wall thicknesses comparable to *Speg*^*+/+*^ mice. Also, the increased LV internal diameter and volume seen in *Speg*^*−/−*^ mice was alleviated by treatment with CPCs, resulting in LV dimensions analogous to wild-type mice (Fig. 10a). These data demonstrate that injection of wild-type CPCs into *Speg*^*−/−*^ mice *in utero* is able to rescue these mice from heart failure in the postnatal period.

Our original report of *Speg*^*−/−*^ mice on a 129Sv × C57BL/6 genetic background did not suggest developmental lethality^10^. In the present study, we assessed live births from breeding of *Speg*^*+/−*^ mice on a pure C57BL/6 genetic background, injected with either vehicle (phosphate buffered saline (PBS), −) or wild-type CPCs (+) expanded in culture. On a pure C57BL/6 background, the per cent of live *Speg*^*−/−*^ births is only 5.4%, instead of the expected Mendelian level of 25% with breeding of *Speg*^*+/−*^ mice (Fig. 10b). This suggests a significant amount of *in utero* death in *Speg*^*−/−*^ mice on a pure C57BL/6 background. Injection of wild-type CPCs at embryonic day 13.5 leads to live births of 27.1% of *Speg*^*−/−*^ pups. These data suggest that injection of wild-type CPCs *in utero* significantly increases the live births of *Speg*^*−/−*^ pups (fivefold), and rescues the offspring from *in utero* death. To determine whether the expression of Speg in CPCs is important for the improved outcome, *Speg* mutant CPCs were injected at embryonic day 13.5 and live births were assessed. Compared with *Speg*^*−/−*^ foetuses receiving wild-type (+/+) CPCs, injection of *Speg* mutant (−/−) CPCs resulted in a significant reduction in live births of *Speg*^*−/−*^ pups (5.7%, Supplementary Fig. 10), which is analogous to the level of live births of *Speg*^*−/−*^ pups receiving vehicle injections (Fig. 10b). These data support the functional abnormalities of *Speg*^*−/−*^ CPCs. Finally, even in the absence of cardiac injections, Speg^−/−^ pups on a C57BL/6 background do not survive beyond postnatal day 1.

## Discussion

MLCK family members are known to play important roles in myocyte function. MLCKs are responsible for myosin regulatory light chain phosphorylation in skeletal and smooth muscle, and this has recently been shown in heart muscle by cardiac MLCK^29^. Phosphorylation of myosin regulatory light chains regulates sarcomere assembly, and subsequent cardiac function^29^,^30^. Knockdown of cardiac-MLCK in zebrafish resulted in immature-appearing sarcomere structures, and the suggestion that cardiac-MLCK may be important for cardiogenesis^30^. We have previously shown that the MLCK family member Speg is upregulated during striated muscle differentiation^8^ and that mice with a disruption in the *Speg* gene locus have a decrease in phosphorylation of the sarcomeric protein α-tropomyosin, and these mice develop a dilated cardiomyopathy^10^. Mutations in the *Speg* gene have also been identified in humans with congenital centronuclear myopathy, and these patients may also present with a dilated cardiomyopathy^11^. Further investigations in *Speg*^*−/−*^ mice showed a decreased number of cardiomyocytes per mm^3^ of myocardium, suggesting an altered generation of cells during development^10^, and immature-appearing cardiomyocytes (Fig. 1). Thus, beyond a role for Speg in fully developed cardiomyocytes, we proposed that Speg would also be critical for the function of CPCs, and for the differentiation of CPCs into mature, functional cardiomyocytes.

Our data demonstrate that CPCs harvested from *Speg*^*−/−*^ mice, compared with *Speg*^*+/+*^ mice, have defects in all properties of progenitor cells. While the overall number of c-kit-positive, lineage-negative cells is not decreased in *Speg*^*−/−*^ hearts (Fig. 3a), mutant CPCs show a marked defect in clone formation (Fig. 3d), a decrease in clone size (Fig. 3b,c) and a reduction in growth potential (Fig. 3e,f). Moreover, the inability of these cells to express sarcomeric α-actin and cTnT under differentiation conditions (Fig. 4), suggests that disruption of the *Speg* gene locus in CPCs leads to a notable block in cardiomyocyte differentiation and subsequent cellular maturation. As seen in Fig. 8, injection of *Speg* mutant CPCs into a *Speg*^*−/−*^ foetus does not result in maturation of the recipient heart to the extent of a *Speg*^*−/−*^ foetus receiving wild-type CPCs. At this time, we are not certain whether this functional defect in the *Speg* mutant CPCs is related to an abnormality in their ability to engraft and survive, or an alteration in the mutant cells to differentiate and interact with surrounding cells or to produce paracrine actions. To our knowledge, this is the first evidence that disruption of a MLCK family member leads to an abnormality in the functional properties of CPCs. Moreover, these defects in CPCs, and altered cardiomyocyte maturation, are associated with the development of a dilated cardiomyopathy (ref. 10 and Figs 6 and 10).

Previously, we have shown that c-kit-positive CPCs, harvested from mice at 16–18 dpc, are able to engraft, asymmetrically divide, acquire a myocyte phenotype and regenerate adult infarcted myocardium in mice^20^. In addition, other investigators have shown that in cardiomyopathic hearts at the time of transplantation, or congenital hearts at the time of surgery, the number of c-kit-positive cells was threefold higher in neonates compared with heart tissue from children >2 years of age^31^. Furthermore, when isolating cardiosphere-derived cells from human neonatal hearts, the cells had a larger population of c-kit-positive cells (also Flk-1- and ISL1-positive cells) and a greater regeneration potential when injected into adult rat hearts after left anterior descending artery ligation, compared with cardiosphere-derived cells from human adult hearts^32^. Taken together, these studies demonstrated that CPCs can be isolated from patients with cardiomyopathies or congenital heart diseases^31^, and that CPCs harvested from foetal or neonatal hearts (mouse and human, respectively)^20^,^32^ have tremendous regenerative potential.

To further understand the importance of CPCs in the *Speg* mutant phenotype, and to comprehend the therapeutic potential of CPCs *in utero*, we investigated whether administering wild-type CPCs into foetuses of *Speg*^*+/−*^ breeding would curtail the development of heart failure in the *Speg* mutant neonates. CPCs injected into foetal hearts at 13.5 dpc showed evidence of engraftment at 19.5 dpc, in the neonatal period after a normal delivery. Confocal imaging revealed that wild-type CPCs were able to differentiate and fully mature in the *Speg*^*−/−*^ hearts (Fig. 9a). In addition, these engrafted wild-type cells appeared to also promote maturation of endogenous mutant cardiomyocytes (Fig. 9b). To strengthen this concept, we repeated injections of wild-type CPCs into foetuses at 13.5 dpc, and on day 1 after birth the hearts were harvested and flow cytometry was performed to assess green fluorescently labelled cells originating from the CPCs, and also for cells staining for cTnT. Quantification of the flow cytometry data revealed that on day 1 after birth, 10.9±0.8% of the total cell population originated from the injected CPCs, while 5.6±0.3% of the total cell population were engrafted cells that had differentiated into cardiomyocytes. Further analyses revealed that 14.2±0.9% of the hearts were composed of cardiomyocytes derived from the exogenous injected CPCs, and 85.8±0.9% of the hearts were composed of endogenous cardiomyocytes. While c-kit-positive cells have classically been shown to directly engraft and regenerate cardiac tissue, it has also been shown that c-kit-positive cardiac stem cells can interact with endogenous cardiomyocytes through gap junctions to exchange microRNAs (mi-499) and improve their differentiation and integration potential^33^. Moreover, cardiospheres have been shown to act through paracrine actions to promote regeneration^34^. Our present data suggest that maturation of the *Speg*^*−/−*^ myocardium in mice receiving wild-type CPCs goes beyond the concept of just CPC engraftment and replacement of the abnormal myocardium. Future studies will focus on mechanisms contributing to this novel maturation process, including the importance of cell-to-cell interactions and paracrine effects of the CPCs on endogenous cardiomyocytes.

To elucidate the potential of cell therapy promoting paracrine effects in the present study, we considered comparing cardiomyoctye maturation in mice receiving injections of CPCs *in utero* with mice receiving cardiac injections of MSCs, which are known to promote many of their actions by paracrine effects. However, MSCs are significantly larger than haematopoietic stem cells^35^, which in tissue are comparable in size with c-kit-positive CPCs. Moreover, it has been shown that a significant decrease in viability occurs when MSCs are passed through narrow-bore needles (25–26 s gauge needle sizes)^36^. Thus, for technical reasons, these experiments were not feasible, as the beveled glass micropipettes used for the micro-ultrasound-guided injection of CPCs *in utero* have a much smaller internal diameter. While the small size of c-kit-positive CPCs allowed for these cells to be injected, the larger MSCs lodged in the beveled glass micropipettes and did not pass into the hearts when injections were attempted.

Interestingly, assessment of the recipient hearts revealed that overall engraftment of exogenously injected CPCs did not differ between wild-type and *Speg* mutant hearts (Fig. 9c and Supplementary Fig. 7). However, the CPCs that engrafted in dysfunctional *Speg* mutant hearts promoted myocardial regeneration of both RV and LV walls (throughout the RV wall and subendocardial regions of the LV and septum), and alleviated the negative remodelling of the ventricular walls (Fig. 6). This was further confirmed by echocardiography, as administration of CPCs *in utero* was able to improve LV function of *Speg*^*−/−*^ hearts, and normalize LV wall thickness and chamber dimensions (Fig. 10). The appearance and function of *Speg* mutant hearts after receiving CPCs was analogous to wild-type hearts on day 1 after birth. This improvement in cardiac function was also associated with a significant increase in live births of *Speg*^*−/−*^ mice after receiving wild-type CPCs. However, this increase in live births of *Speg*^*−/−*^ mice was totally lost when the foetuses were injected with *Speg* mutant CPCs, demonstrating the importance of Speg for CPC function *in vivo* (Supplementary Fig. 10). While cellular therapy with mesangioblast progenitor cells has been shown to prevent a dilated cardiomyopathy in a model of Duchenne muscular dystrophy in mice^37^, administration of cells was performed 5–6 weeks after birth. To our knowledge, the present study provides the first demonstration that cardiac injection of CPCs *in utero* is able to mitigate heart failure after birth.

We also observed that a subpopulation of cells engrafted, but remained in an undifferentiated state, and formed a putative niche (Fig. 5g–i). These findings allow us to speculate regarding the therapeutic potential of CPCs when administered *in utero*. The ability of the CPCs to engraft, differentiate and to promote maturation of endogenous cardiomyocytes explains why CPCs can provide more acute therapeutic benefit. However, the potential of CPCs engrafting and forming putative niches provides a rationale for long-term therapy. Progenitor cell niches offer an environment where CPCs remain in an undifferentiated state, connected to supporting cells in the myocardium^26^. Asymmetrical division of CPCs allows for these cells to generate daughter CPCs (permitting replenishment of the CPC pool) and daughter committed cells that contribute to myocardial regeneration under pathophysiologic conditions^26^,^38^,^39^. We believe that the formation of putative niches by wild-type CPCs in the myocardium of *Speg* mutant hearts may allow for a source of normal CPCs in these dysfunctional hearts, which contain abnormal endogenous CPCs. Further investigation into the long-term benefit of *in utero* CPC administration in *Speg* mutant mice will help to elucidate the potential of CPC therapy for long-term outcome in newborn diseases.

At the present time, foetal cardiac intervention (FCI) in humans is a therapeutic tool that has been used to treat conditions in which there is high risk for prenatal or neonatal death, or diseases that will likely result in major cardiac morbidity^40^. In these circumstances, the goal of FCI is to improve survival or to improve cardiac growth, development and function so as to alter a detrimental postnatal outcome. FCI procedures are carried out using a percutaneous ultrasound-guided approach in which a needle is used to gain foetal access, and mechanical interventions are performed for congenital cardiovascular anomalies^40^. With recent evidence that CPCs^41^,^42^ and cardiosphere-derived cells^43^ have therapeutic benefit in human adult patients with ischaemic cardiomyopathy or after myocardial infarction, respectively, we postulate that *in utero* cell therapy may have future implications for the treatment of foetal cardiovascular diseases with high risk for postnatal morbidity and mortality.

## Methods

### *Speg* mutant mice

*Speg*^*−/−*^ (mutant) mice were previously generated as described^10^, and backcrossed nine consecutive generations to yield *Speg*^*−/−*^ mice on a pure C57BL/6 genetic background. Since the *Speg*^*−/−*^ mice die shortly after birth, all studies were performed on the offspring of *Speg*^*+/−*^ (heterozygous) breeding. Thus, *Speg*^+/+^ (wild type) mice were littermates of *Speg*^*−/−*^ mice.

### Adipocyte differentiation

Mouse CPCs (*Speg*^+/+^ and *Speg*^−/−^) and mouse MSCs (harvested from adipose tissue) were cultured until confluent, and then placed in adipose differentiation medium (Lonza). Every 3 days the medium was changed between adipose differentiation medium and maintenance medium, for a total of 14 days. On day 14, the cells were fixed and stained with Oil Red O (Sigma-Aldrich) as described^1^,^2^.

### Isolation and characterization of mouse CPCs

For the cells used in the *in utero* injections, hearts from three litters of day 1 newborn pups, each litter from a different *Speg*^*+/−*^ dam, were harvested as described below. The cells were kept separate, and genotyping was performed to identify *Speg*^*+/+*^ and *Speg*^*−/−*^ CPCs. Thus, for the *in vivo* studies, three independent harvests of CPCs were used. Cells used for the *in vitro* studies were not only from these harvests, but also from the hearts of additional day 1 newborn pups from litters of *Speg*^*+/−*^ dams. To acquire an adequate number of cells, the majority of studies were performed on CPCs that were expanded in culture, and characterized prior to use. The experiments performed on c-kit-positive lineage-negative cells that were not expanded in culture are shown in Fig. 3a, as the flow cytometry analyses were completed on freshly isolated cells.

CPCs were quantitated from day 1 mouse hearts (*Speg*^*+/+*^ or *Speg*^*−/−*^) by enzymatic dissociation in a solution containing collagenase I, 10 mg ml^−1^ and collagenase II, 25 mg ml^−1^ (Worthington Biochemical). The cells (≈1.5–2.5 × 10^6^) were incubated with anti-mouse c-kit antibody conjugated with PECy7 (eBioscience) and FITC anti-mouse Lineage Cocktail (BioLegend), and then sorted by flow cytometry for c-kit-positive, lineage-negative cells. For *in vitro* experiments, cells harvested after enzymatic dissociation were cultured for 1–2 weeks in growth medium containing DMEM/F12 medium (Lonza) supplemented with foetal bovine serum (FBS, 10%, Invitrogen), murine basic fibroblast growth factor (20 ng ml^−1^, PeproTech), murine epidermal growth factor (20 ng ml^−1^, PeproTech), insulin/transferrin/sodium selenite (Biowittaker) and leukaemia inhibitor factor (LIF, 10 ng ml^−1^, Chemicon). The CPCs were then isolated with anti-mouse c-kit (CD117) MicroBeads (Miltenyi Biotec), and lineage depletion performed by incubating the cells with the Mouse Haematopoietic Progenitor Cell Enrichment Kit (STEMCELL technologies) and collecting the negative fraction. Early passage CPCs were characterized by FACS to demonstrate the presence of c-kit, and to document their undifferentiated state. For antibodies see Supplementary Table 3.

To assess the differentiation potential of CPCs, cells were cultured in specialized differentiation medium for 7 days and assayed by immunocytochemistry, flow cytometry and qRT-PCR (see sections below). For cardiomyocyte differentiation, CPCs were cultured for 7 days in DMEM/F12 medium supplemented with reduced FBS (2%), BMP 4 (50 ng ml^−1^, PeproTech), TGF-β2 (10 ng ml^−1^, PeproTech) and an absence of LIF. For smooth muscle cell differentiation, CPCs were cultured for 7 days in Medium 231 with Smooth Muscle Differentiation Supplement (Invitrogen, S0085) and FBS (5%), and for endothelial cell differentiation CPCs were cultured in Clonetics Endothelial Cell Medium (Lonza, CC-3156). To differentiate cells in a lineage unbiased manner, LIF was removed from the CPC growth medium and dexamethasone (10 nM) was added.

### Flow cytometry and immunocytochemistry

For characterization of CPCs, cells were cultured in either growth medium or differentiation medium, washed with PBS and detached using a non-enzymatic solution HyQTase (Thermo Fisher Scientific). Cells suspensions were pre-incubated with rat IgG_2b_ anti-mouse CD16/CD32 monoclonal antibody (BD Bioscience) at room temperature for 15 min prior to staining with specific antibodies or isotype matched control antibodies. CPCs were incubated with the first antibodies conjugated with fluorescence at 4 °C for 30 min, or incubated with the first antibody followed by a second antibody conjugated with fluorescence at 4 °C for 30 min. If the target protein was intracellular, the cells were fixed and then permeabilized prior to staining. To establish whether CPCs were lineage negative, cells were fixed and labelled for c-kit and haematopoietic markers. In addition, the expression of proteins by immunocytochemistry and/or flow cytometry for cardiomyocytes (Nkx2.5, cTnT sarcomeric α-actin), smooth muscle cells (GATA6, calponin and smooth muscle α-actin) and endothelial cells (Ets1, vWF, CD31 and Flk-1) was determined. For antibodies, see Supplementary Table 3. Stained cells were washed with PBS and analysed using a BD FACS Canto II. At least 10,000 events were collected and data were analysed using FlowJo software.

### Assessment of c-kit-positive, lineage-negative cells in heart tissue

Hearts from *Speg*^*+/+*^ and *Speg*^*−/−*^ pups, 1 day after birth, underwent enzymatic dissociation. Red blood cells were lysed, followed by counting of the remaining cells. The cells were stained with anti-mouse c-kit antibody conjugated with PECy7 and FITC anti-mouse Lineage Cocktail, followed by flow cytometric analysis. C-kit-positive, lineage-negative cells were calculated as a percentage of the total cells in the hearts.

### Limiting dilution and clone formation

CPCs were plated at 100 cells per 100 mm diameter dish (low density, ≈1 cell per 60 mm^2^) to obtain multicellular clones derived from a single founder cell. At day 10, 20 and 40, clones were stained with crystal violet. The dishes were photographed for colony counts. The clones with >50 cells were counted. To assay the number of cells per clone, we harvested clones using cloning cylinders and counted the cell numbers with a Beckman cell counter. Please note that we were unable to assess clonality by plating a single CPC into wells of Terasaki plates because CPCs from *Speg*^*−/−*^ hearts were unable to survive sorting by FACS.

### Growth analysis

For the studies assessing CPC growth, the same number of cells harvested from *Speg*^*+/+*^ or *Speg*^*−/−*^ mice were plated, and cells were then counted daily for 5 days or BrdU uptake into nuclei was assessed on day 5 (Roche, BrdU Labelling and Detection Kit).

### qRT-PCR

Total RNA was isolated from mouse CPCs, either before or after muscle differentiation, using Trizol reagent (Invitrogen), and reverse transcription was performed using the SuperScript III First-Strand Synthesis System (Invitrogen). QRT-PCR was performed using the Sybr green kit (Bio-Rad) in a StepOnePlus Real-time PCR System (Invitrogen). Primers for mouse cTnT, MEF-2C, GATA6, VEZF1 and GAPDH were purchased from Invitrogen. Expression of mouse cTnT, MEF-2C, GATA6 and VEZF1 were normalized to GAPDH expression levels. QRT-PCR was also performed for Speg and Nkx2.5 using TaqMan probes, and normalized to GAPDH (Invitrogen). The RT-PCR reaction was performed using TaqMan Fast Universal PCR Mix kit (Invitrogen) with 6-carboxyfluorescein fluorophore. A summary of the quantitative RT-PCR primers is provided in Supplementary Table 4.

### Transmission electron microscopy

Hearts from 18.5-dpc foetuses were fixed in 0.1 M sodium cacodylate buffer containing 2% paraformaldehyde and 2.5% glutaraldehyde for 2 h at room temperature and then overnight at 4 °C. The ventricular tissue was cut into thin strips and then subdivided into small cubes constituting a randomized sampling of the thickness of the ventricular wall. The foetuses were postfixed in 1% osmium tetroxide/1.5% potassium ferrocyanide, dehydrated and embedded in Epon/Araldite resin as previously described^10^. The blocks were then thin-sectioned for analysis by electron microscopy.

Quantitation of the ultrastructural composition of cardiomyocytes from *Speg*^*+/+*^ and *Speg*^*−/−*^ hearts by transmission electron microscopy was performed using a square array of 140 sampling points of intersecting lines as described^44^. The number of points lying over specific structures (myofibrils, mitochondria, nuclei or undifferentiated cytoplasm) was calculated as a percentage of the volume composition of cardiomyocytes.

### Immunohistochemistry

Hearts from mouse foetuses at 18.5 dpc or newborn mouse pups at 19.5 dpc (day 1 after birth) were arrested in diastole by direct intra-cardiac injections of cadmium chloride (0.1 M). The hearts were then formalin fixed, dehydrated and embedded in paraffin. Sections (5 μm) were then stained for analysis. Evaluation of heart tissue was performed by light and confocal microscopy by targeted fluorescent antibodies as described previously^20^,^45^. Fluorescent staining was performed with antibodies targeting cTnT, sarcomeric α-actin, Speg and c-kit, and also CPCs labelled with green fluorescent dye PKH67 (Sigma-Aldrich) was assessed by confocal imaging. For antibodies see Supplementary Table 3.

### Quantitation of cardiomyocyte size

Sections (5 μm) of hearts from *Speg*^*+/+*^ and *Speg*^*−/−*^ mice at day 1 after birth (receiving either no injections or wild-type CPCs at 13.5 dpc) were immunostained for cTnT (cardiomyocytes), DAPI (nuclei) and Wheat Germ Agglutinin (cell border). Random images of cardiomyocytes from the LV, RV and septum were captured using a fluorescence microscope. We then measured the shortest diameter across the nucleus of individual cells using ImageJ by an investigator blinded to the groups, and the cross-sectional area of the cells was calculated^46^,^47^ (300 cells per location, in each group).

### Injections of CPCs *in utero*

Injections of CPCs were performed using the Vevo 2100 high-resolution micro-ultrasound system and a 50-MHz probe (VisualSonics, and the Vevo Integrated Rail System II with Injection System). Timed pregnancies of *Speg*^*+/−*^ mice were performed to allow injections at 13.5 dpc. The genotypes of the foetuses were not known at the time of the injections, thus all foetuses from a specific pregnancy were injected with *Speg*^*+/+*^ CPCs labelled with the green fluorescent dye PKH67, which incorporates into the membrane of cells and is equally distributed to daughter cells when they divide, or *Speg*^*+/+*^ CPCs harvested from transgenic mice expressing enhanced GFP under the direction of the human ubiqutin C promoter (C57BL/6-Tg(UBC-GFP)30Scha/J, Jackson Laboratories). Foetuses were also injected with *Speg*^*−/−*^ CPCs. For the injection procedure, the dam was anaesthetized, a laparotomy performed, the uterine horn exposed in a sterile manner and injections were performed directly through the uterus into the walls of the LV, RV and septum using a beveled glass micropipette. Each location was injected twice, with 2,500 cells in a volume of 70 nl PBS per injection. The uterine horn was then placed back into the abdomen and a normal delivery of the pups occurred 6 days later. Supplementary Table 5 provides an overview of the number of pregnant dams studied, the number of foetuses injected and the number of live births. The difference between the number of injected foetuses and the live births are deaths that occurred *in utero*. The Harvard Medical Area Standing Committee on Animals, Harvard Medical School, approved animal care and use for all experiments in this study.

In a group of newborn pups that had received injections of green fluorescently labelled CPCs at 13.5 dpc, their hearts were harvested on day 1 after birth, and enzymatically digested into single-cell suspensions. Flow cytometry was then performed to assess the percentage of green fluorescently labelled cells, and also for cells staining for cTnT, from the total population of cardiac cells.

### Echocardiography

A Vevo 2100 high-resolution micro-ultrasound system and a 50-MHz probe were used for transthoracic echocardiography on non-anaesthetized newborn pups (day 1 after birth). The hearts were imaged in the two-dimensional parasternal short-axis view, and an M-mode echocardiogram of the mid-ventricle was recorded at the level of papillary muscles^10^,^48^. Wall thickness in systole and diastole (LVPW,s; LVPW,d; IVS,s; and IVS,d), and the end-systolic and end-diastolic internal dimensions of the LV (LVID,s; LVID,d) and the end-systolic and end-diastolic volume of the LV (LV vol,s; LV vol,d) were measured from the M-mode image.

(1) FS %=(LVID,d—LVID,s)/LVID,d × 100;

(2) EF %=(LV vol, d—LV vol,s)/LV vol,d × 100.

Echocardiograms were performed on all of the pups from a pregnancy, and genotyping was performed later. Thus, the echocardiograms were performed in a blinded manner, without prior knowledge of the genotype.

### Statistical analysis

For comparisons between two groups, we used Student's two-tailed unpaired *t*-test, or Mann–Whitney *U*-testing for non-parametric analyses where the data were not normally distributed. For the analysis of CPC proliferation, the rate of growth was derived by fitting the data to the exponential growth curve, and then comparisons between groups were then made by Student's unpaired *t*-test. For comparisons of more than two groups, either a two-way analysis of variance (ANOVA) or a one-way ANOVA (followed by Bonferroni's or Newman–Keuls post test) was used, depending on the number of comparisons performed. For comparison of mortality between groups, Fisher's exact test was used. Statistical significance for all comparisons was accepted at *P*<0.05. The numbers of samples per group (*n*), or the numbers of experiments, are specified in the figure legends.

## Additional information

**How to cite this article:** Liu, X. *et al*. Rescue of neonatal cardiac dysfunction in mice by administration of cardiac progenitor cells *in utero*. *Nat. Commun.* 6:8825 doi: 10.1038/ncomms9825 (2015).

## Supplementary Material

Supplementary Information Supplementary Figures 1-10, Supplementary Tables 1-5 and Supplementary References.
