# Supplementary material for: Rescue of neonatal cardiac dysfunction in mice by administration of cardiac progenitor cells in utero
Source: Nat Commun. 2015 Nov 23;6:8825. doi: 10.1038/ncomms9825 (PMC4673493; doi:10.1038/ncomms9825)
Supplement: Supplementary Information — Supplementary Figures 1-10, Supplementary Tables 1-5 and Supplementary References. [file ncomms9825-s1.pdf]

Supplementary Figure 1. Clone of Speg$^{+/+}$ CPC

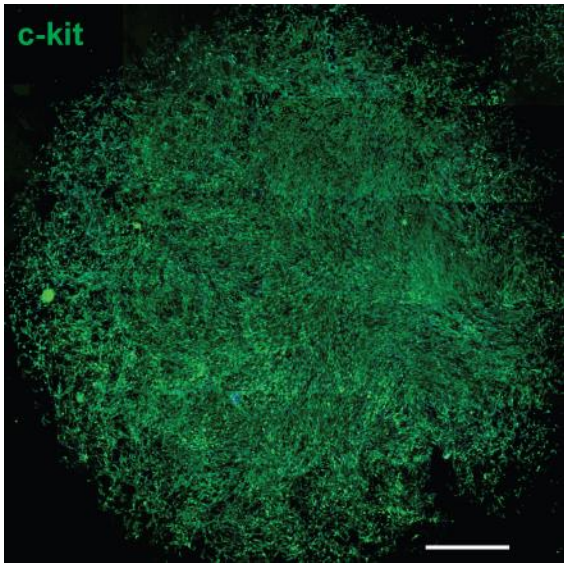

A clone originating from a putative CPC harvested from the heart of a Speg$^{+/+}$ mouse. The clone was immunostained for c-kit (green) and DAPI (blue). The scale bar represents 1000 µm.

Supplementary Figure 2. Expression of Speg in CPCs

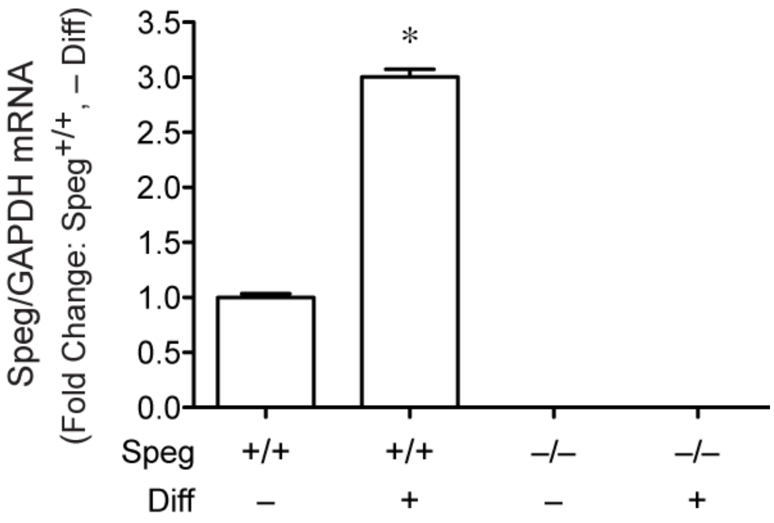

qRT-PCR was performed for Speg on RNA extracts from Speg$^{+/+}$ (white bars) and Speg$^{–/–}$ (black bars) CPCs after culture in medium to retain the cells in an undifferentiated state (–Diff), or in medium to promote cardiomyocyte differentiation (+Diff). Expression levels of Speg mRNA were divided by expression levels of the control gene GAPDH, and shown as a fold change in expression compared with Speg+/+ –Diff. Data are presented as mean ± SEM, n=3 independent experiments per group. * P<0.0001 versus Speg$^{+/+}$ –Diff, Speg$^{–/–}$ –Diff, and Speg$^{–/–}$ +Diff using One-way Analysis of Variance, followed by Newman-Keuls multiple comparison test.

Supplementary Figure 3. Characterization of CPCs

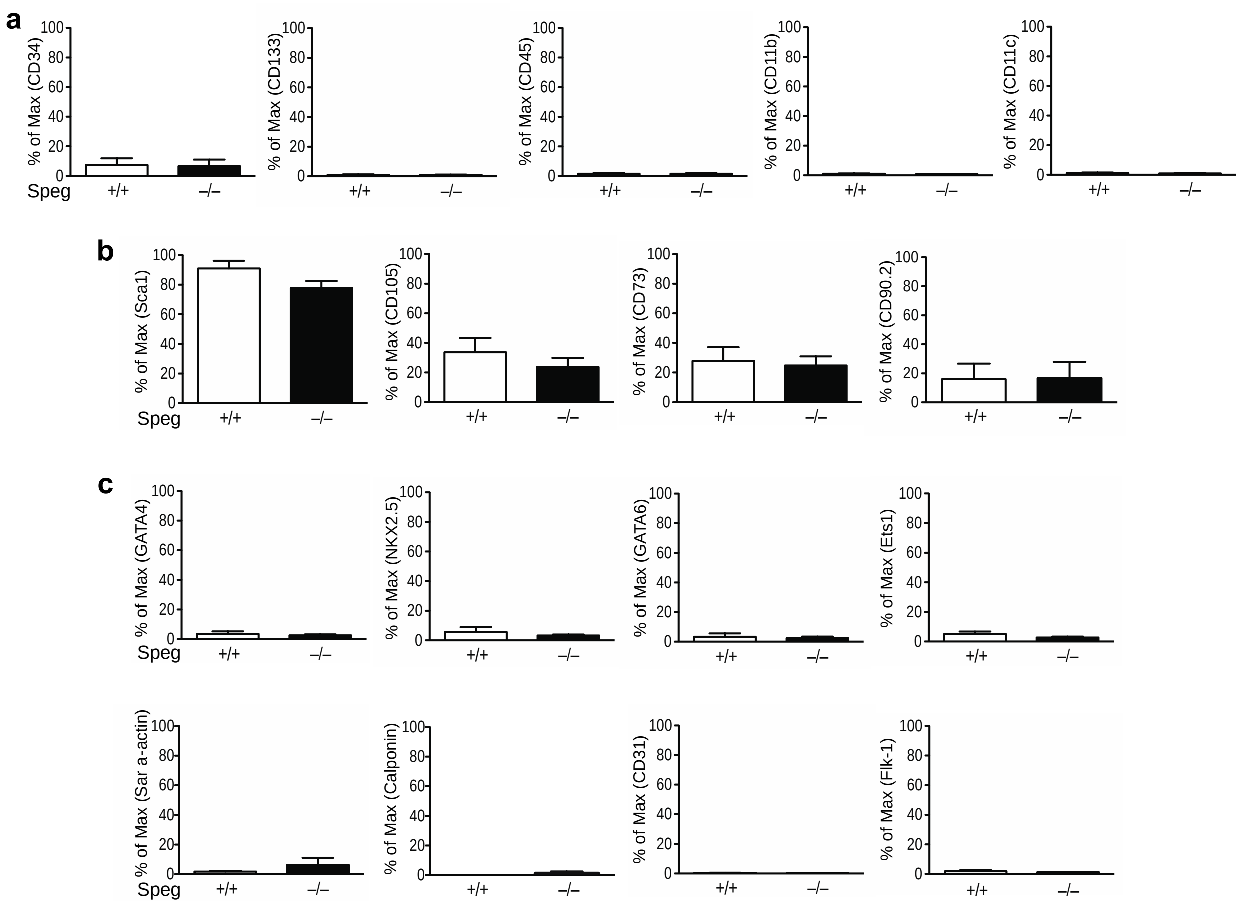

Flow cytometry characterization of Speg$^{+/+}$ (white bars, cells from a minimum of 3-4 different harvests) and Speg$^{-/-}$ (black bars, cells from a minimum of 2 different harvests) c-kit-positive putative CPCs. (**a**) Cells were assessed for markers of hematopoietic lineage including CD34, CD133, CD45, CD11b, and CD11c. (**b**) Cells were assessed for Sca1, and for markers of mesenchymal cells including CD105, CD73, and CD90.2. (**c**) Cells were assessed for markers of CPC commitment (GATA4), or transcription factors and cytoplasmic markers of cardiomyocyte (NKX2.5, Sar α-actin), smooth muscle (GATA6, Calponin), and endothelial (Ets1, CD31, Flk-1) cell lineages.

Supplementary Figure 4. Putative CPCs do not differentiate into adipocytes

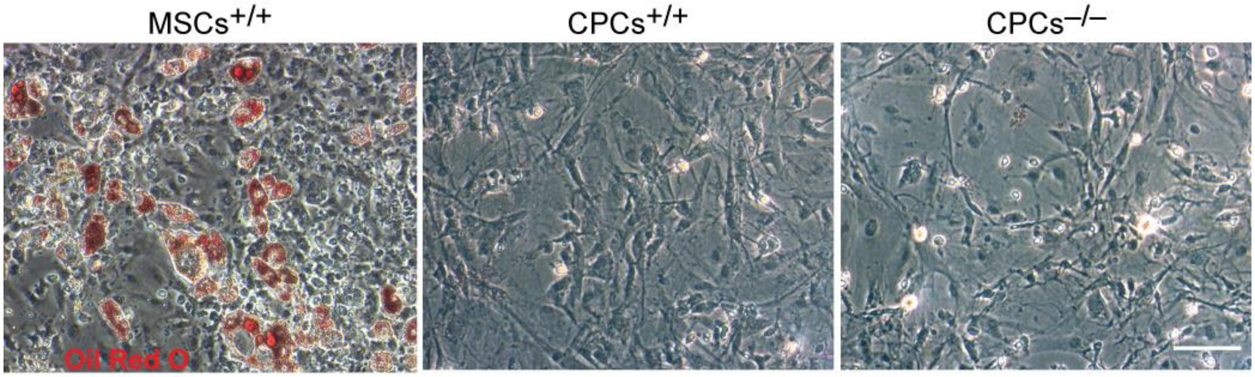

MSCs harvested from mouse adipose tissue, and CPCs harvested from hearts of Speg$^{+/+}$ and Speg$^{–/–}$ mice, were cultured in adipogenic differentiation medium for 14 days, and the cells were stained with Oil Red-O. Fat droplets stain red in cells that differentiate into adipocytes. Representative images of control MSCs (left panel), Speg$^{+/+}$ CPCs (middle panel) and Speg$^{–/–}$ CPCs (right panel). The scale bar represents 100 µm.

Supplementary Figure 5. Exogenous CPCs engraft and differentiate into mature cardiomyocytes in Speg$^{-/-}$ hearts

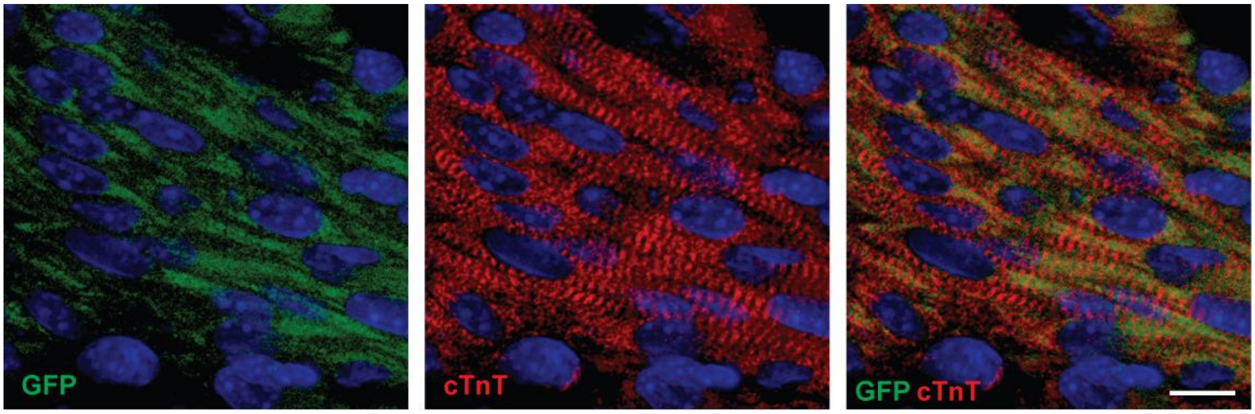

CPCs were harvested from hearts of C57BL/6-Tg(UBC-GFP)30Scha/J mice and isolated by fluorescence-activated cell sorting (FACS). CPCs were injected *in utero* at 13.5 dpc into hearts of fetuses from Speg$^{+/-}$ breeding. The hearts were harvested at day 1, and sections from a Speg$^{-/-}$ heart were immunostained for cardiac troponin T (cTnT). The confocal images show green fluorescent protein (GFP) for exogenous cells (green, left panel), the cardiomyocyte marker cTnT (red, middle panel), and merged images showing double positive cells (right panel). The scale bar represents 10 µm.

Supplementary Figure 6. Gating strategy for flow cytometry

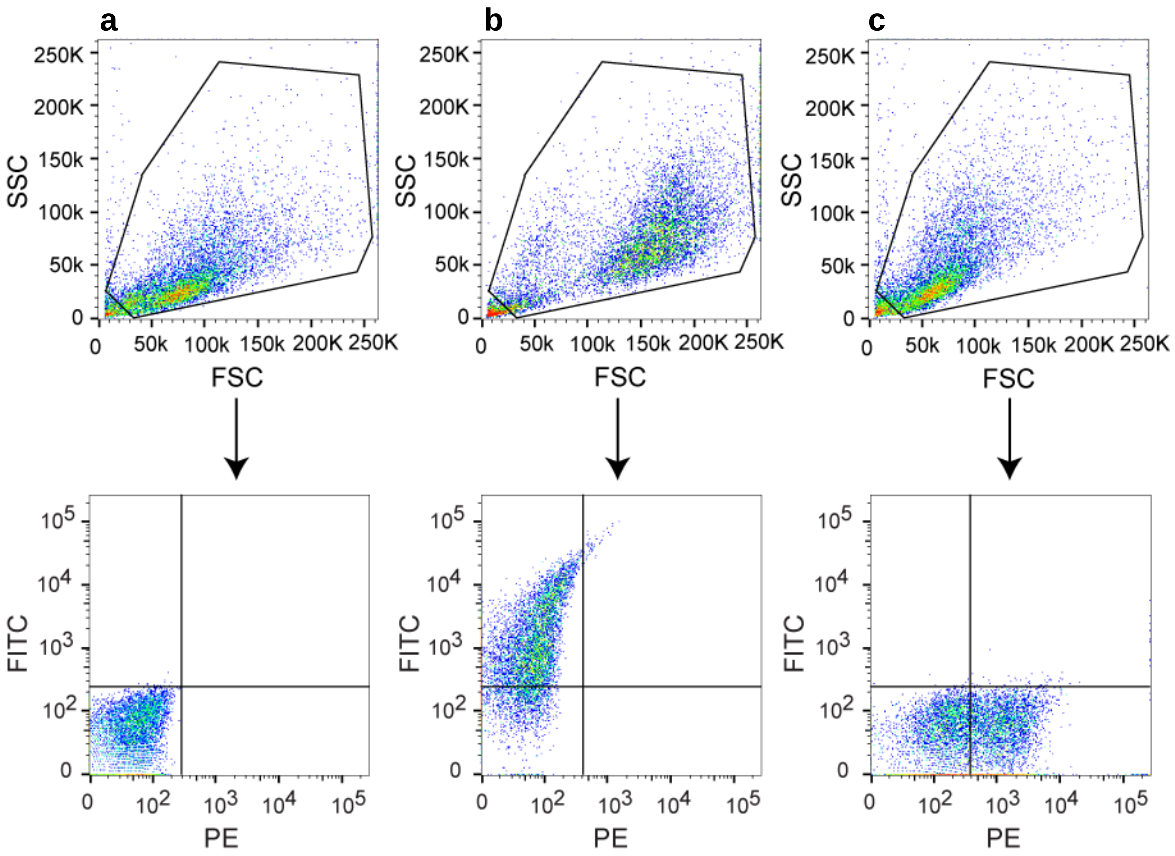

Cells were initially gated for forward and side scatter (**a**-**c**, upper panels). Next, unstained cells from hearts not injected with CPCs were gated as negative controls for FITC and PE (**a**, lower panel). To gate for cells positive for FITC (green fluorescence), we analyzed CPCs dyed with PKH67 (**b**, lower panel). Finally, to gate for cells positive for PE (cTnT), we analyzed cells harvested from newborn hearts not injected with CPCs and stained for cTnT (**c**, lower panel).

Supplementary Figure 7. Percentage of total cardiac cells originating from exogenous CPCs, and percentage of total cells differentiating into cardiomyocytes

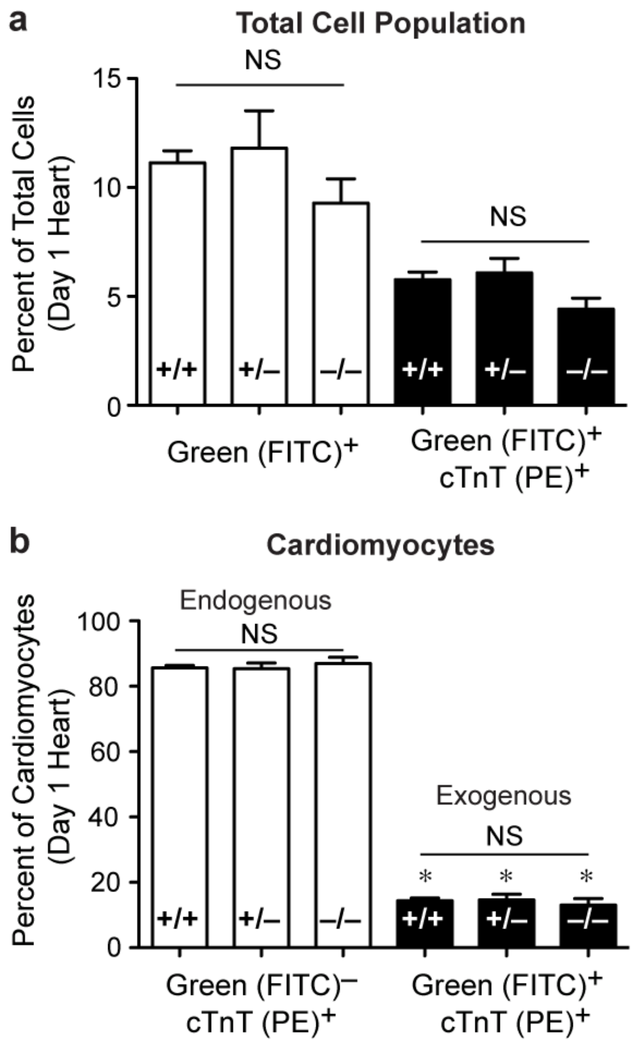

(**a**) Percentage of total cells that are green (white bars), or green cells expressing cTnT (black bars). Each group is broken down into genotypes of cells from Speg mice (wild-type, n=7, +/+; heterozygous, n=8, +/–, and homozygous mutant, n=5, –/–).
(**b**) Percentage of cTnT positive cardiomyocytes that are not green (endogenous, white bar) or green (exogenous, black bar). Each groups is broken down into genotypes of cells from Speg mice (wild-type, n=7, +/+; heterozygous, n=8, +/–, and homozygous mutant, n=5, –/–). P<0.0001; * versus Endogenous cardiomyocytes using One-way Analysis of Variance, followed by Newman-Keuls multiple comparison test.

Supplementary Figure 8. Effect of CPCs on the percentage of endogenous cardiomyocytes

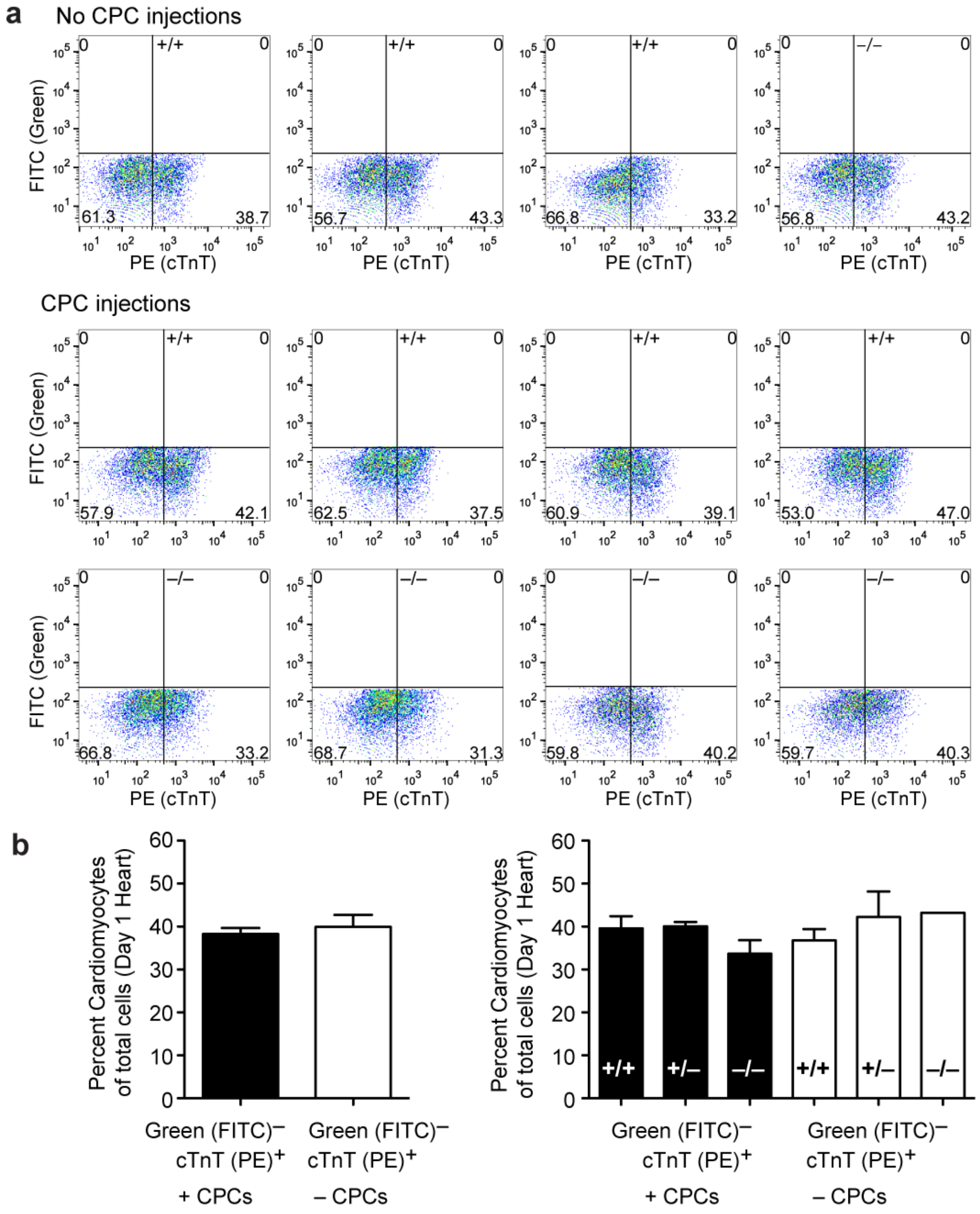

(**a**) Gating for FITC-negative cells only. Upper panels show representative flow cytometry pseudo-color density plots (4 plots from a total of 9 fetal hearts [2 pregnant dams] with no CPC injections, 8 plots from a total of 20 fetal hearts [3 pregnant dams] with CPC injections) of cells harvested from wild-type (+/+) and Speg mutant (–/–) hearts at day 1, assessing cells expressing cTnT (PE). (**b**) The left panel demonstrates the percentage of endogenous cells of hearts injected with CPCs (+) that are positive for cTnT (black bar, n=20), or endogenous cells from hearts not injected with CPCs (–) that are positive for cTnT (white bar, n=9). The right panel

separates the two groups (cTnT positive, with or without CPC injections) into genotypes (wild-type, +/+; heterozygous Speg, +/–; homozygous Speg mutant, –/–).

Supplementary Figure 9. Diastolic assessment of Speg$^{-/-}$ and Speg$^{+/+}$ hearts after *in utero* injections of CPCs

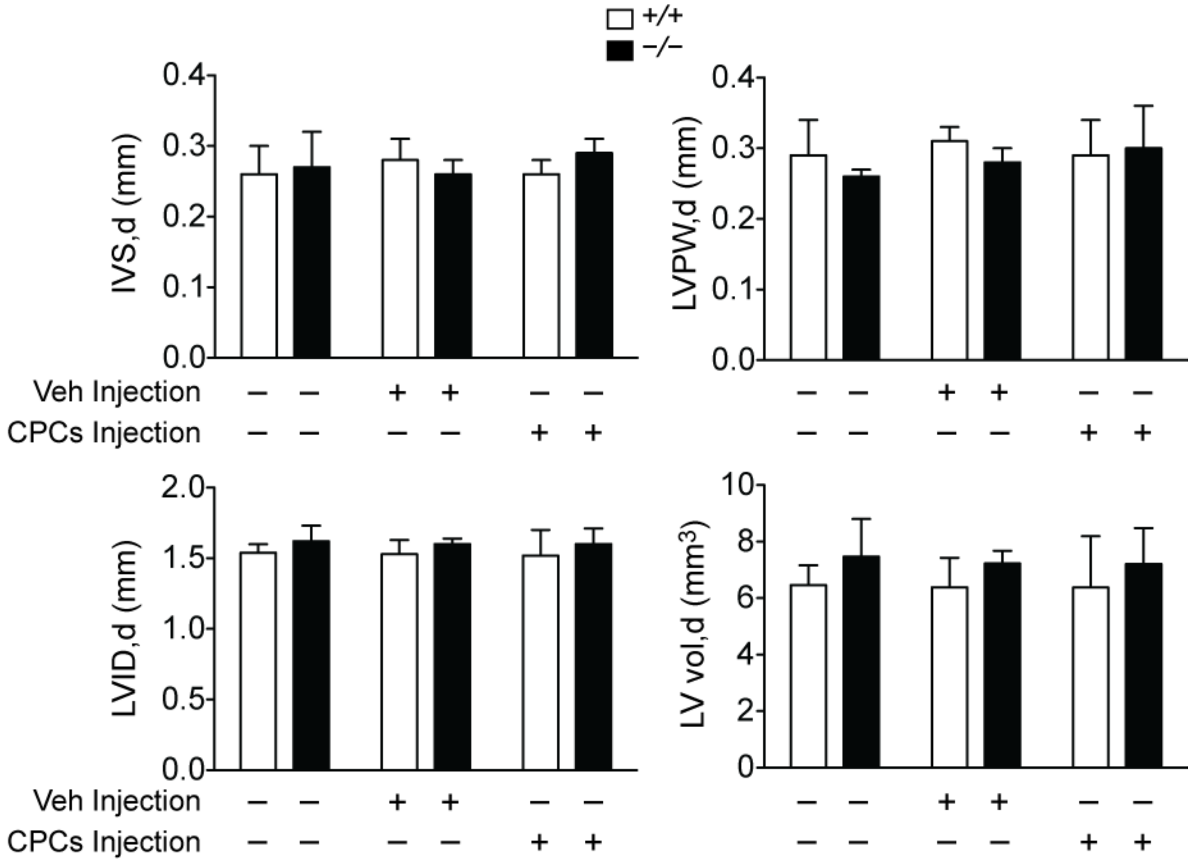

Speg$^{+/+}$ (white bars, n=6-7/group) and Speg$^{-/-}$ (black bars, n=4-5/group) mice received either no injections (–), or intra-cardiac injections (+) with vehicle (Veh) or CPCs at 13.5 dpc. On day 1 after birth (19.5 dpc), echocardiograms were performed to assess cardiac function. Measurements for thickness of the intraventricular septum (IVS) and LV posterior wall (LVPW), and dimensions of the LV internal diameter (LVID) and LV volume (LV vol) were performed during diastole (d). Data are presented as mean±SEM.

Supplementary Figure 10. Live births of Speg$^{-/-}$ neonates is not improved by injection of mutant CPCs

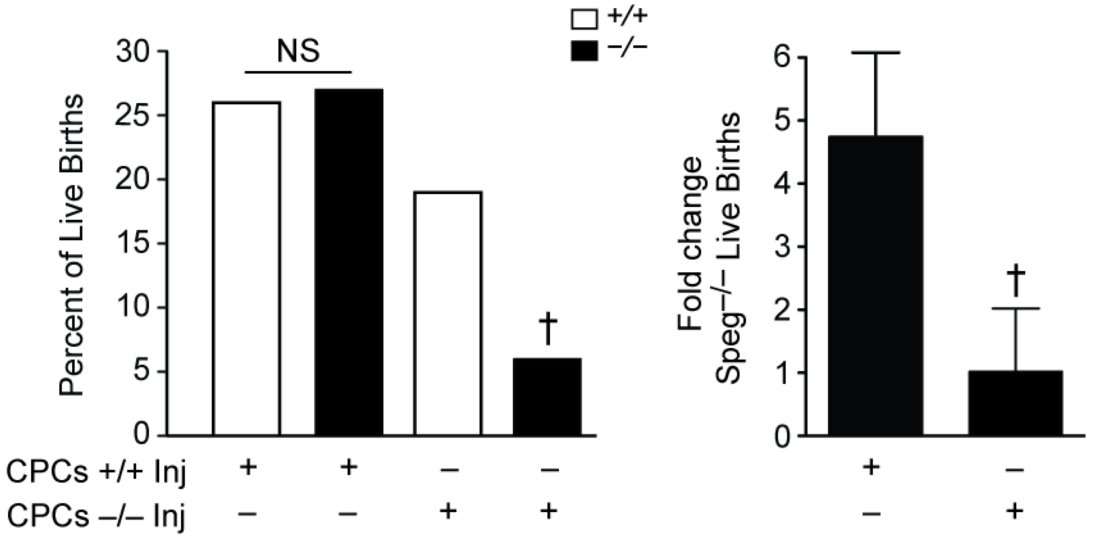

Fetuses of Speg$^{+/-}$ pregnant dams were injected with either wild-type CPCs (+/+, n=6 litters) or mutant CPCs (–/–, n=5 litters) at 13.5 dpc. On day 1 after birth (19.5 dpc), percent of live births were assessed in Speg$^{+/+}$ (white bars) and Speg$^{-/-}$ (black bars) pups (left panel). *P*=0.0291; † versus Speg$^{-/-}$ pups, wild-type CPCs injection using Fisher's exact test. In the right panel, fold change (mean ±SEM) in live births of Speg$^{-/-}$ pups receiving either wild-type (+/+) or mutant (–/–) CPCs. *P*=0.0262; † versus Speg$^{-/-}$ pups, +/+ CPCs injection using Student's unpaired t test.

Supplementary Table 1. Percentage of total cardiac cells originating from CPCs (FITC+, green), and percentage of total cells differentiating into cardiomyocytes (PE+, cTnT)

	% FITC+ PE–	% FITC+ PE+	% FITC– PE+	% FITC– PE–	% FITC+ cells of total cells	% FITC+ PE+ cells of total cells	% FITC– PE+ of total PE+ cells (% Endogenous Myocytes)	% FITC+ PE+ of total PE+ cells (% Exogenous Myocytes)
**Dam 1, Inj CPCs (705)**								
Pup +/+	5.1	4.8	29.6	60.6	9.8	4.8	86.1	13.9
Pup +/+	6.8	4.6	22.7	65.9	11.3	4.6	83.2	16.8
Pup +/–	3.8	4.1	32.5	59.6	7.9	4.1	88.9	11.1
Pup +/–	6.6	7.2	30.4	55.9	13.8	7.2	80.9	19.1
Pup –/–	6.2	4.1	20.6	69.2	10.2	4.1	83.4	16.6
Pup –/–	6.5	5.7	28.6	59.3	12.1	5.6	83.5	16.5
Pup –/–	5.4	4.7	28.2	61.7	10.1	4.7	85.8	14.2
Pup –/–	3.5	5.0	36.5	54.9	8.5	5.0	88.0	12.0
**Dam 2, inj CPCs (756)**								
Pup +/+	7.0	6.6	36.5	50.0	13.6	6.6	84.8	15.2
Pup +/+	5.7	6.3	30.8	57.1	12.1	6.3	82.9	17.1
Pup +/–	1.7	2.7	36.5	59.1	4.4	2.7	93.0	7.0
Pup +/–	11.3	8.0	30.5	50.1	19.3	8.0	79.2	20.8
Pup +/–	6.0	6.3	35.5	52.1	12.3	6.3	84.9	15.1
Pup +/–	8.7	7.7	32.5	51.2	16.3	7.7	80.9	19.1
**Dam 3, inj CPC (784)**								
Pup +/+	5.9	5.5	37.3	51.3	11.4	5.5	87.3	12.7
Pup +/+	3.5	5.7	44.4	46.4	9.2	5.7	88.6	11.4
Pup +/+	3.5	7.0	44.0	45.4	10.6	7.0	86.2	13.8
Pup +/–	4.9	7.3	43.0	44.8	12.2	7.3	85.5	14.5
Pup +/–	2.8	5.4	46.9	44.9	8.2	5.4	89.7	10.3
Pup –/–	2.8	2.7	42.4	52.1	5.5	2.7	94.0	6.0
**Mean**					**10.9**	**5.6**	**85.8**	**14.2**
**SEM**					**0.8**	**0.3**	**0.9**	**0.9**

Supplementary Table 2. Effect of CPCs on the percentage of endogenous cardiomyocytes (FITC [green]–, PE+ [cTnT])

	% FITC– PE+	% FITC– PE–	% FITC– PE+ of total FITC– cells (% Endogenous Myocytes)
**Dam 1, Inj CPCs (705)**			
Pup +/+	36.2	63.8	36.2
Pup +/+	26.4	73.6	26.4
Pup +/–	36.5	63.5	36.5
Pup +/–	36.8	63.2	36.8
Pup –/–	23.3	76.7	23.3
Pup –/–	33.2	66.8	33.2
Pup –/–	31.3	68.7	31.3
Pup –/–	40.3	59.7	40.3
**Dam 2, Inj CPCs (756)**			
Pup +/+	42.1	57.9	42.1
Pup +/+	37.5	62.5	37.5
Pup +/–	39.5	60.5	39.5
Pup +/–	39.1	60.9	39.1
Pup +/–	40.5	59.5	40.5
Pup +/–	40.7	59.3	40.7
**Dam 3, Inj CPC (784)**			
Pup +/+	39.1	60.9	39.1
Pup +/+	47.0	53.0	47.0
Pup +/+	48.8	51.2	48.8
Pup +/–	45.4	54.6	45.4
Pup +/–	42.3	57.7	42.3
Pup –/–	40.2	59.8	40.2
**Mean**			**38.3**
**SEM**			**1.4**

	% FITC– PE+	% FITC– PE–	% FITC– PE+ of total FITC– cells (% Endogenous Myocytes)
**Dam 1, no inj (749)**			
Pup 1 +/+	33.2	66.8	33.2
Pup 2 +/+	31.9	68.1	31.9
Pup 3 +/–	32.8	67.2	32.8
Pup 4 +/–	31.2	68.8	31.2
**Dam 2, no inj (718)**			
Pup 3 +/+	38.7	61.3	38.7
Pup 6 +/+	43.3	56.7	43.3
Pup 5 +/–	54.6	45.4	54.6
Pup 7 +/–	50.2	49.8	50.2
Pup 4 –/–	43.2	56.8	43.2
**Mean**			**39.9**
**SEM**			**2.8**

Supplementary Table 3. Antibodies used for Flow Cytometry Phenotyping and Immunostaining

Epitope	Company/Cat#	Catalog	Host Animal	Concentration/Dilution	Labeling
CD34	BioLegend	128609	Hamster	0.2 mg/ml, 1:50	Direct PE
CD133	BioLegend	141207	Rat	0.2 mg/ml, 1:50	Direct APC
CD45	BioLegend	103112	Rat	0.2 mg/ml, 1:50	Direct APC
CD105	BioLegend	120413	Rat	0.2 mg/ml, 1:50	Direct APC
Flk-1	BD Biosciences	555308	Rat	0.2 mg/ml, 1:50	Direct PE
CD11c	BD Biosciences	557401	Hamster	0.2 mg/ml, 1:50	Direct PE
CD11b	eBioscience	12-0112	Rat	0.2 mg/ml, 1:50	Direct PE
CD31	eBioscience	12-0311	Rat	0.2 mg/ml, 1:50	Direct PE
CD73	eBioscience	12-0731	Rat	0.2 mg/ml, 1:50	Direct PE
Sca1	eBioscience	17-5981	Rat	0.2 mg/ml, 1:50	Direct APC
CD90.2	eBioscience	11-0903	Rat	0.2 mg/ml, 1:50	Direct FITC
c-kit (CD117)	eBioscience	25-1171	Rat	0.2 mg/ml, 1:10	Direct PE/Cy7
c-kit (CD117)	R&D Systems	AF1356	Goat	0.1 mg/ml, 1:10 *	Indirect FITC (Jackson ImmunoResearch #705-096-147 1.5mg/ml, 1:100) *
Speg	Hsieh et al, J Biol Chem, (Ref #8)		Rabbit	1:50 *	Indirect Alexa 555 (Abcam #ab150074, 2mg/ml, 1:500) *
GATA4	Abcam	ab86371	Mouse	1 mg/ml, 1:50	Indirect Alexa 647 (Abcam ab150107, 2mg/ml, 1:50)
GATA6	Abcam	ab22600	Rabbit	1 mg/ml, 1:50	Indirect Alexa 647 (Abcam ab150075, 2mg/ml, 1:50)
Ets1	Abcam	ab10936	Mouse	Tissue culture super, 1:50	Indirect Alexa 647 (Abcam ab150107, 2mg/ml, 1:50)
Cardiac Troponin T	Abcam	ab125266	Rabbit	0.5 mg/ml, 1:50	Indirect Alexa 647 (Abcam ab150075, 2mg/ml, 1:50)
				0.5 mg/ml, 1:200 *	Indirect Alexa 555 (Abcam #ab150074, 2mg/ml, 1:500) *
Cardiac Troponin T	Abcam	ab8295	Mouse	2 mg/ml, 1:50	Indirect Alexa 647 (Abcam ab150107, 2mg/ml, 1:50)
				2 mg/ml, 1:200 *	Indirect Alex 488 (Abcam #ab150105, 2mg/ml, 1:500) *
Calponin	Abcam	ab46794	Rabbit	Tissue culture super, 1:100 *	Indirect Alexa 555 (Abcam #ab150074, 2mg/ml, 1:500) *
Calponin	Sigma-Aldrich	C2687	Mouse	0.5~1 mg/ml, 1:50	Indirect Alexa 647 (Abcam ab150107, 2mg/ml, 1:50)
Sarcomeric a-actin	Sigma-Aldrich	A2172	Mouse	0.6~1.2 mg/ml, 1:200 *	Indirect TRITC (Jackson #715-026-029, 1.5mg/ml, 1:200) *
NKX2.5	Santa Cruz	sc-8697	Goat	0.2 mg/ml; 1:50	Indirect FITC (Invitrogen A11055, 2mg/ml, 1:50)
Wheat Germ Agglutinin	Invitrogen	W11261	N/A	1 mg/ml, 1:200 *	Direct Alex 488 *
vWF	Millipore	AB7356	Rabbit	1 mg/ml, 1:50	Indirect Alexa 647 (Abcam ab150075, 2mg/ml, 1:50)

* Antibodies for immunofluorescent staining
All other antibodies for flow cytometry

Supplementary Table 4. Summary of Primers for Real-time PCR Analysis

Mus cTnT	Forward<br>Reverse	5'- CCTCAAGACCTGTGTGCAGT -3'<br>5'- CCTCTTGCTCTTCCTGTTCC -3'
Mus MEF-2C	Forward<br>Reverse	5'- CTGTCTGGCTTCAACACTGC -3'<br>5'- TAGTGCAAGCTCCCAACTGA -3'
Mus GATA6	Forward<br>Reverse	5'- AAGCGCGTGCCTTCATCAC -3'<br>5'- GAGCCACTGCTGTTACCGGA -3'
Mus VEZF1	Forward<br>Reverse	5'- CCAGGGAAGCAGGTAGAGACAC -3'<br>5'- TTTGACATAGTCCCAGACGACACAG -3'
Mus GAPDH	Forward<br>Reverse	5'- CCTGGAGAAACCTGCCAAG -3'<br>5'- AGGAGACAACCTGGTCCTCA -3'
Mus Speg	Probe	TaqMan®
Mus Nkx2.5	Probe	TaqMan®
Mus GAPDH	Probe	TaqMan®

Supplementary Table 5. Summary of Cardiac Progenitor Cell Injections into Pregnant Dams (Embryonic Day 13.5)

**Speg$^{+/-}$ Dams**	**Total Dams**	**Total injected fetuses (E13.5)**	**Live Births (postnatal day 1)**
Speg$^{+/+}$ CPCs injected	9	67	63
Speg$^{-/-}$ CPCs injected	6	37	30
PBS injected	6	51	45
No injection	7	0	51
**Totals**	28	155	189

Supplementary References

1. Hall SR, Tsoyi K, Ith B, Padera RF, Jr., Lederer JA, Wang Z*, et al.* Mesenchymal stromal cells improve survival during sepsis in the absence of heme oxygenase-1: The importance of neutrophils. *Stem Cells* 2013, **31**(2)**:** 397-407.

2. Zhu H, Guo ZK, Jiang XX, Li H, Wang XY, Yao HY*, et al.* A protocol for isolation and culture of mesenchymal stem cells from mouse compact bone. *Nat Protoc* 2010, **5**(3)**:** 550-560.
